# A Novel Cranial Bone Transport Technique Repairs Skull Defect and Minimizes Brain Injury Outcome in Traumatic Brain Injury Rats

**DOI:** 10.1002/advs.202504467

**Published:** 2025-05-31

**Authors:** Shanshan Bai, Xuan Lu, Xu Yan, Han Su, Yuejun Lin, Zhaowei Jiang, Zhixian Zong, Haixing Wang, Leo Yik Chun Yan, Xiaoting Zhang, Ming Wang, Zhengmeng Yang, Jiakang Jin, Yaofeng Wang, Wayne Yuk‐Wai Lee, Xiaohua Jiang, Ho Ko, Lu Feng, Micky D. Tortorella, Sien Lin, Gang Li

**Affiliations:** ^1^ Stem Cells and Regenerative Medicine Laboratory Li Ka Shing Institute of Health Sciences The Chinese University of Hong Kong Prince of Wales Hospital Shatin Hong Kong SAR 999077 P. R. China; ^2^ Center for Locomotor System Regenerative Medicine and Technology Institutes of Biomedicine and Biotechnology Shenzhen Institute of Advanced Technology Chinese Academy of Sciences University Town of Shenzhen Shenzhen 518055 P. R. China; ^3^ Musculoskeletal Research Laboratory Department of Orthopaedics & Traumatology Faculty of Medicine The Chinese University of Hong Kong Prince of Wales Hospital Shatin Hong Kong SAR 999077 P. R. China; ^4^ Centre for Regenerative Medicine and Health Hong Kong Institute of Science & Innovation Chinese Academy of Sciences Hong Kong Hong Kong SAR 999077 P. R. China; ^5^ Department of Neurosurgery First Hospital of Jilin University Changchun 130000 P. R. China; ^6^ Division of Neurology Department of Medicine and Therapeutics & Li Ka Shing Institute of Health Sciences Faculty of Medicine The Chinese University of Hong Kong Shatin Hong Kong SAR 999077 P. R. China; ^7^ Key Laboratory for Regenerative Medicine of the Ministry of Education of China, School of Biomedical Sciences, Faculty of Medicine The Chinese University of Hong Kong Hong Kong SAR 999077 P. R. China

**Keywords:** cranial bone transport, cranioplasty, meningeal lymphatic drainage, neuro‐inflammation, traumatic brain injury

## Abstract

TBI (traumatic brain injury) is a major cause of mortality and morbidity among young adults with limited therapeutic strategies. Cranial bone transport (CBT) technique is a safe, less invasive, and relatively simple surgical technique in bone reconstruction, which has been used to repair cranial bone defects and deformity corrections. The current studies are to determine the effects of CBT surgery on cranial bone regeneration as well as neurological functional recovery in TBI. CBT treatment alleviated lesion size and promoted learning, motor, and memory recovery in TBI rats. The meningeal lymphatic drainage function is enhanced, evidenced by increased intake of ovalbumin conjugated with Alexa Flour 647(OVA‐A647) in meningeal lymphatic vessels (MLVs) and deep cervical lymph nodes (dCLNs). CBT accelerated P‐tau clearance while decreasing Iba1 induced neuroinflammatory response in TBI rats. Notably, improvement of CBT treatment is significantly abolished by the ablation of MLVs via MAZ51, a small‐molecule inhibitor primarily targeting vascular endothelial growth factor receptor‐3 (VEGFR‐3). Furthermore, after bone transport treatment, bone regeneration in the CBT sites continued consolidation, bone defects in TBI are replaced with new bone more quickly after CBT surgery. Taken together, the study is a proof‐of‐concept de‐novo study to prove CBT can significantly improve the outcomes of brain recovery and cranioplasty in TBI rats.

## Graphical abstract

This study introduces a novel, safe, and effective surgical technique: Cranial bone transport (CBT) to improve traumatic brain injury (TBI) outcomes in rats. CBT significantly accelerated skull defect bone repair in addition to its promoting effects on neurological function recovery. This work provides an alternative therapy for patients suffering from TBI, especially for those with decompressive craniectomy.

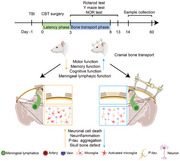

## 1 Introduction

Traumatic brain injury (TBI) is a brain injury caused by sudden external mechanical force. As a major cause of death and disability globally, TBI adds a substantial burden on society and families worldwide.^[^ ^1^, ^2^ ^]^ The primary and secondary injuries after TBI lead to complicated pathophysiological processes, involving a series of biological events triggered during this period, for example, apoptosis, neuroinflammation, brain edema, and mitochondrial dysfunction.^[^ ^3^, ^4^ ^]^ Additionally, TBI also develops neurodegeneration in the brain. It is reported that TBI increases the risk of Alzheimer's disease, Parkinson's disease, and dementia.^[^ ^5^ ^]^ Accumulation of tau, amyloid beta, and TAR‐DNA‐binding protein‐43 may persist for months and years after TBI.^[^ ^6^ ^]^ Recent evidence showed that ablation of meningeal lymphatic drainage function before TBI induces exacerbated neuroinflammation and cognitive deficits, indicating maintenance of the function of meningeal lymphatic vessels (MLVs) is essential for TBI outcome.^[^ ^7^ ^]^ Moreover, impairment of the lymphatic system induced the tau, especially phosphorylated‐tau (P‐tau) aggregation in the brain as early as several days after TBI.^[^ ^8^ ^]^ Collectively, these findings suggest that targeting MLVs and lymphatic function may provide a new strategy for TBI therapy, and accelerating P‐tau clearance via meningeal lymphatic function may be an alternative way to alleviate brain injury in TBI.

Decompressive craniectomy is widely used for TBI patients with intracranial hypertension, which is achieved by removing a large bone flap and dura to reduce brain swelling and intracranial pressure.^[^ ^9^, ^10^ ^]^ However, those patients are required to undergo a second surgery called cranioplasty for the restoration of skull integrity. The cranioplasty protects the brain from exposure through bone defects not only for cosmetic reasons but also for physiological reasons.^[^ ^11^ ^]^ Although synthetic material can get excellent cosmetic effects and contouring ability, the autologous graft is used as the gold standard due to its decreased infection rate and lack of host immune response.^[^ ^12^, ^13^ ^]^ Cranial bone transport (CBT) has previously been applied to reconstruct calvarial bone defects in plastic and reconstructive surgery.^[^ ^14^, ^15^ ^]^ As a safe and less invasive surgical method, the CBT procedure could successfully repair cranial defects with no additional costs and immunological rejection concerns, in contrast to the titanium mesh and other biomaterials for cranial bone defect repair, the therapeutic effects of CBT in cranioplasty for TBI need to be fully investigated.

CBT is a kind of employing external fixation technology and has been developed for calvarial bone defects therapy for many years.^[^ ^16^, ^17^ ^]^ The mainstay of bone transport owing to mechanical transduction, which applied to the bone segment and gap affects cellular activity profoundly, during this process, continuous tension stretch stimulation derived from the moving bone segment promotes angiogenesis, osteogenesis and tissue regeneration to repair the bone defect.^[^ ^18^, ^19^ ^]^ Technically, the procedure of CBT is simple and safe, the dura and brain are intact during treatment. Furthermore, we have proved that CBT promotes brain recovery in the ischemic stroke rat model through improving angiogenesis, neurogenesis, and meningeal lymphatic function in our previous publication.^[^ ^20^ ^]^ Ischemic stroke and TBI share many common pathological processes despite different etiology, including reduced oxygen and blood supply, brain edema, breakdown of the blood‐brain barrier, and elevated neuroinflammation.^[^ ^21^, ^22^ ^]^ Based on the positive findings in our middle cerebral artery occlusion (MCAO) rat model studies, we propose that CBT may also enhance functional recovery in TBI conditions perhaps through modulation of MLVs drainage as well.

In the present study, to directly investigate the therapeutic potential of CBT in neurological function recovery and skull defect repair caused by TBI, we first proved that CBT showed a significant reduction of lesion size of TBI and dramatically increased the viable neurons, and CBT also reversed cognitive, memory impairment and motor function deficits in the TBI rats. Furthermore, CBT treatment alleviated MLVs dysfunction after TBI, further reducing neuroinflammation and increasing P‐tau clearance in brain trauma. In contrast, these beneficial effects were reversed partially when the blockade of the VEGF‐C/VEGFR‐3 pathway by MAZ51, Notably, skull defects were evaluated after the CBT bone consolidation period (≈2 months) in rats, the results demonstrated increased newly formed bone formation after CBT and histological sections revealed elevated bone matrix deposition and increased expression of osteogenic markers. In conclusion, we propose that CBT is a potential therapeutic method for enhancing brain recovery and cranioplasty in TBI management.

## 2 Results

### 2.1 CBT Reduced Neuronal Injury and Improved Neurological Deficits in TBI Rats

**Figure 1:**
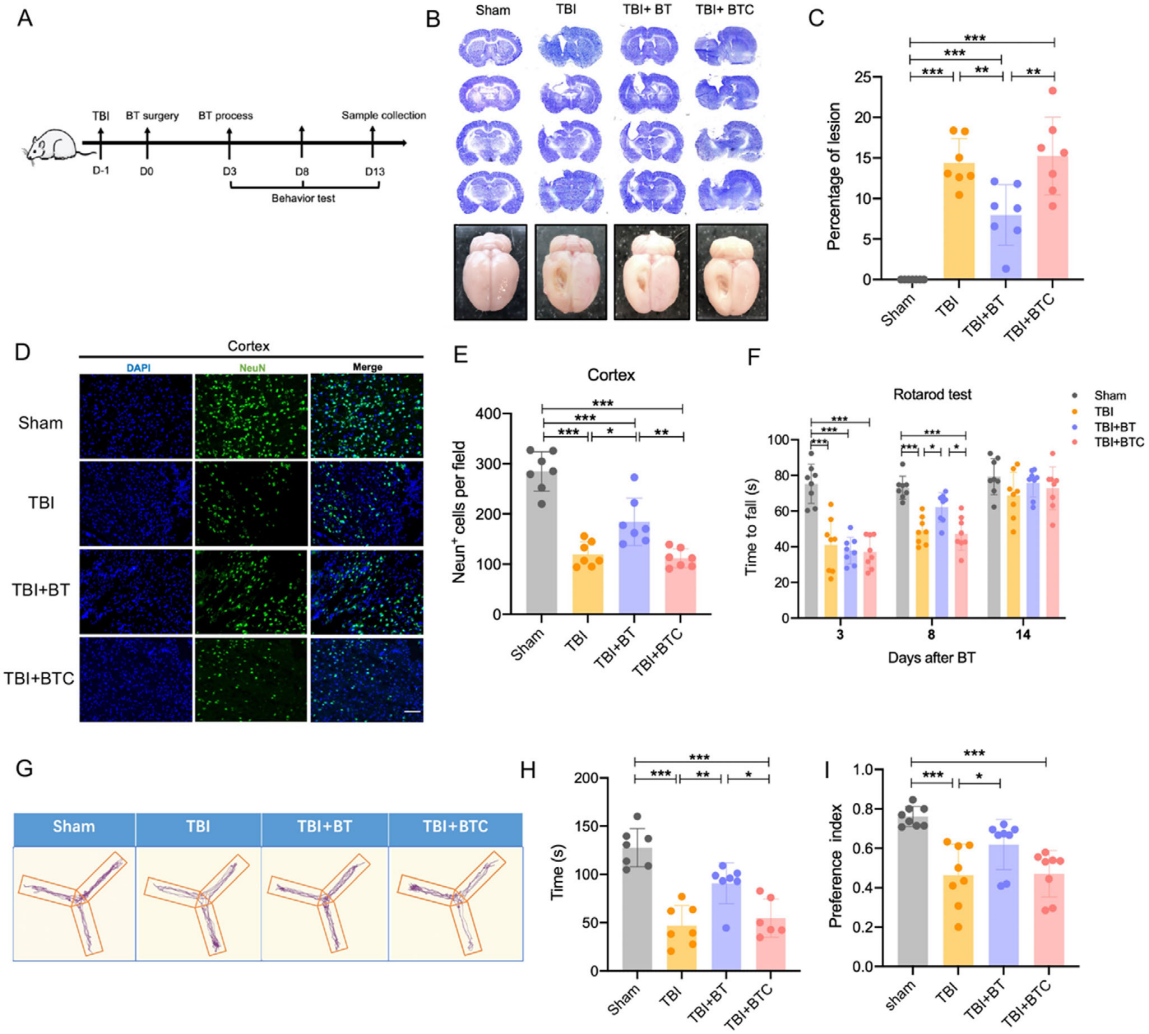
CBT reduced lesion size, attenuated neuronal death, and improved motor, memory, cognitive function in TBI rats. A) Schematic diagram of experiment design. B) Representative Nissl staining and images of gross view of four groups. C) Quantification of percentage of lesion size, calculated by ratio of the lesion area in ipsilateral brain to whole area of contralateral brain. D) Representative image of perilesional cortex section showing NeuN+ immunofluorescence at 14 days after TBI, scale bar = 100 µm. E) Quantification of total viable neurons in the perilesional cortex. F) Time to fall from the rotating rod of TBI rats significantly increased after 8 days of BT surgery. G) Representative images of track in spatial memory test part for four groups. H) Histograms represent the duration in B arm for each experimental group. I) Histograms represent the preference index for each experimental group. Preference index = time spent with novel object/total exploration time. ^*^ *p* < 0.05, ^**^ *p* < 0.01, ^***^ *p* < 0.001. Data are shown as mean ± SD.

To investigate the therapeutic effects of CBT, CBT surgery was performed 24 h after the establishment of TBI model, bone transport was conducted for 10 days followed by a latency of 3 days (**Figure** **1**A). Nissl staining was used to assess the injury in the brain 14 days post‐TBI. While the brains in the Sham group were intact, Nissl sections and gross view images in the other three groups demonstrated varying degree of injury and altered morphology in the ipsilateral brain (Figure 1B). Quantification results showed a significant reduction of lesion size in the CBT group (Figure 1C), In addition, we used immunofluorescence staining to check the neuronal cell survival 10 days after CBT treatment at different regions in the lesioned hemisphere of the brain. We measured the number of viable neurons (NeuN+ cells) in the ipsilateral cortex and striatum and calculated the percentage area of NeuN+ positive cells in hippocampus 14 days after TBI. The results revealed that TBI caused significant neuronal loss in the cortex, striatum, and hippocampus. While the number of neurons in cortex of TBI+ bone transport (TBI+BT) group was significantly increased than that of the TBI group (Figure 1D,E). There was also a slight increase in the striatum but no significance (Figure S1A,C, Supporting Information). The neurons in the hippocampus were dense, therefore it is inaccurate to measure the number of neurons, the proportion area of NeuN+ positive cells were calculated, and results showed that CBT group has dramatically increased the viable neurons in the hippocampus (Figure S1B,D, Supporting Information). Animal behavioral tests, including rotarod test, Novel object recognition (NOR) test and Y maze were performed to evaluate functional recovery during this period. The results indicated that TBI rats with bone transport achieved better motor functional improvements. The time to fall off was significantly increased compared to untreated TBI rats at 8 days after CBT surgery (Figure 1F). In Y maze test, the spontaneous alternation part showed that there was a decrease in all TBI groups compared to the Sham group, CBT didn't show any beneficial effects (Figure S1E, Supporting Information). However, the movement track of the TBI+BT group was mainly concentrated in the novel arm (Figure 1G), significant difference was seen in the duration in the spatial memory test part of Y maze test between the TBI and TBI+BT groups (Figure 1H), Significant differences were also found in the preference index and discrimination index in the NOR test between the TBI and TBI +BT groups (Figure 1I; Figure S1F, Supporting Information). The above evidence suggests that CBT alleviated brain injury improved neurological deficits in TBI rats.

### 2.2 CBT Treatment Enhances Meningeal Lymphatic Function and P‐Tau Clearance in TBI Rats

**Figure 2:**
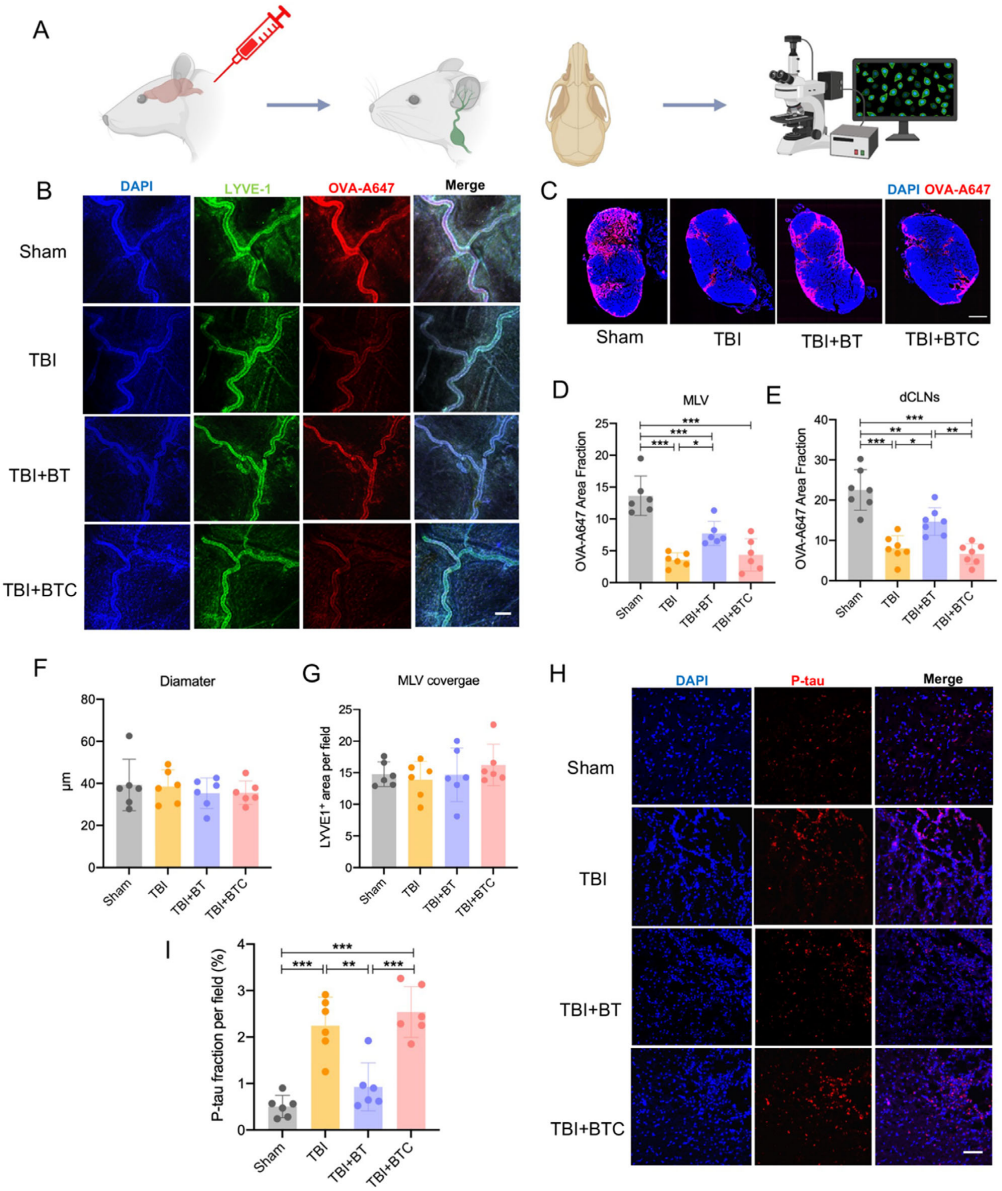
Augmentation of meningeal lymphatic drainage after CBT in TBI rats. A) Schematic of the experimental layout. Rats were injected intra‐cisterna magna (i.c.m.) with OVA647, then dCLNs and brains were collected after 2 h. B) Representative images of MLVs in middle meningeal artery region with OVA‐A647 (red) stained with DAPI (blue), scale bar = 100 µm. C) Representative images of dCLNs with OVA‐A647 (red) stained with DAPI (blue), scale bar = 500 µm. D) Quantification of OVA‐A647 in MLVs in four groups. E) Quantification of OVA‐A647 in dCLNs in four groups. F) Quantification of diameter of MLVs in the four groups. G) Quantification of MLVs coverage in four groups. H) Representative images of perilesional cortex stained with P‐tau (red) and DAPI (blue), scale bar = 50 µm. I) Quantification of P‐tau fraction in four groups. *p<0.05, **p<0.01, ***p<0.001. Data are shown as mean ± SD.

In recent years, emerging evidence has shown the crucial role of MLVs in TBI conditions. Previous studies demonstrated that TBI induced impairments of meningeal lymphatic drainage which exacerbated neuroinflammation and neurological deficits.^[^ ^7^ ^]^ Additionally, tau accumulation in TBI has been proved by lots of studies, and the hyperphosphorylation tau can be observed in brain as early as the first day after TBI,^[^ ^23^, ^24^ ^]^ and impairment of lymphatic drainage function may lead to P‐tau protein accumulation in the TBI brain.^[^ ^8^ ^]^ To investigate whether CBT could migrate meningeal lymphatic drainage deficits in TBI rats and enhance P‐tau clearance, the meningeal drainage function was evaluated by injecting ovalbumin conjugated with Alexa Flour 647 (OVA‐A647) tracer to cisterna magna (**Figure** **2**A), and analysis of deep cervical lymph nodes (dCLNs) revealed that the OVA‐A647 amount was significantly reduced in the TBI group compared to the Sham group but the amount was increased in the CBT group 14 days post‐TBI, indicating that CBT treatment improved the meningeal lymphatic drainage function in TBI rats (Figure 2B‐E). However, the diameter of MLVs and LYVE‐1+ area was not significantly different among the groups, suggesting that CBT treatment did not enhance the MLVs coverage (Figure 2F,G). Moreover, P‐tau aggregation was associated with meningeal lymphatic drainage function, immunofluorescent staining results showed that TBI significantly elevated P‐tau level in the ipsilateral cortex, which was in agreement with previous publications^[^ ^25^, ^26^ ^]^ and the CBT treatment dramatically reduced P‐tau level compared to the TBI group (Figure 2H,I), suggesting the enhanced meningeal lymphatic drainage function (increased P‐tau clearance) after CBT treatment.

### 2.3 CBT Alleviated TBI‐Induced Neuroinflammation

**Figure 3:**
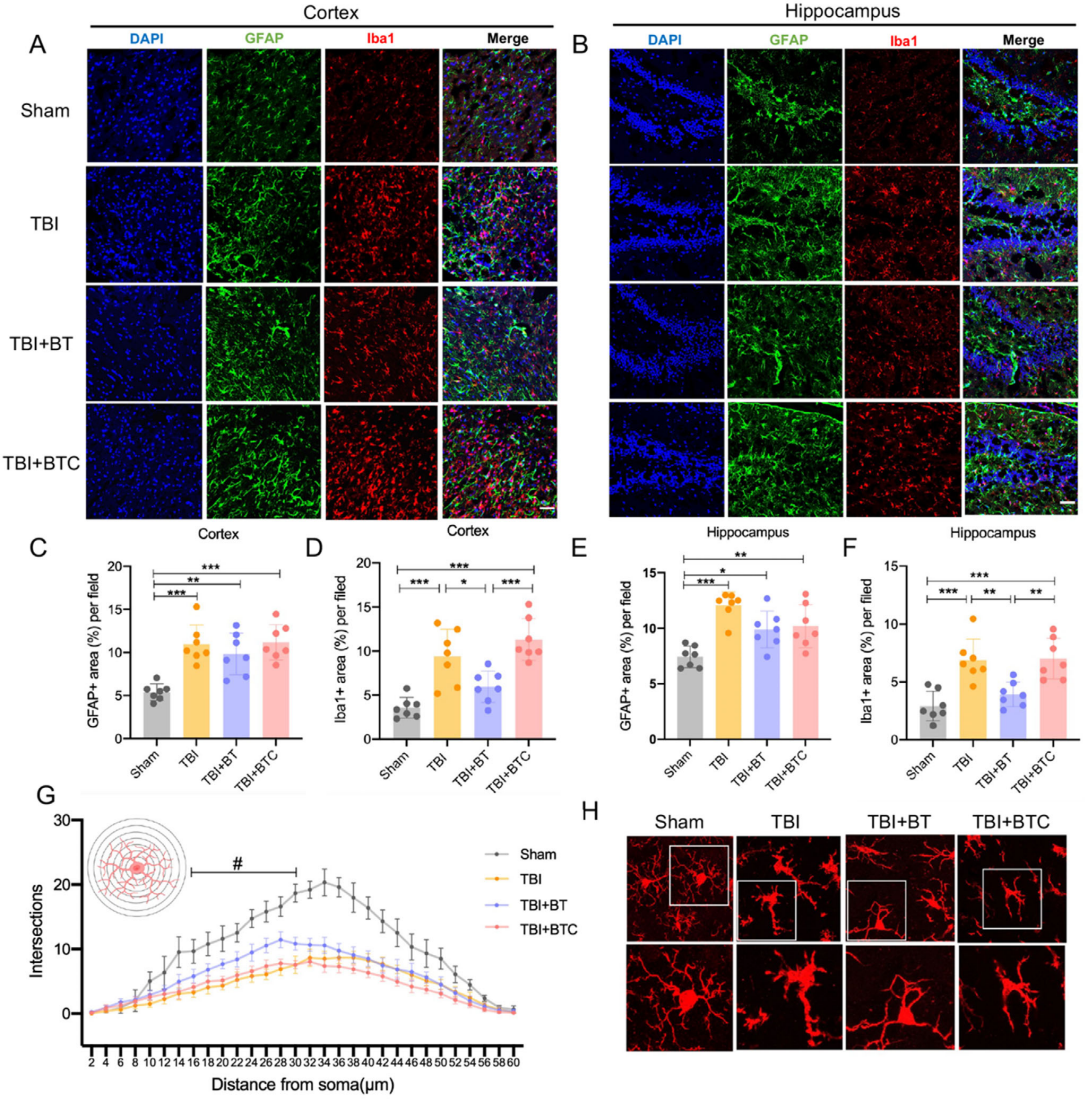
CBT alleviated TBI‐induced neuroinflammation and microglia activation. A) Representative images of the cortex region in TBI brain in the four groups, scale bar = 50 µm. B) Representative images of the hippocampus region in TBI rat brain in the four groups, scale bar = 50 µm. C‐D) Quantification of the percent area of GFAP (C) and Iba1 (D) immunoreactivity per field of view in the cortex by Image J software. (E‐F) Quantification of the percent area of GFAP E) and Iba1 F) immunoreactivity per field of view in the hippocampus by Image J software. G) Representative reconstructions of morphology of Iba1+ cells. H) Sholl analysis of Iba1+ cells interactions in four groups. (#) indicates that significant differences between TBI and TBI+BT groups. *p<0.05, **p<0.01, ***p<0.001. Data are shown as mean ± SD.

Next, we investigated whether CBT treatment reduces neuroinflammation in TBI rats. Astrocytes are the most abundant cells in brain, which contribute to inflammation responses in TBI through secreting multiple cytokines and chemokines.^[^ ^27^ ^]^ As the resident immune cell population in brain, microglia activation after TBI mediates neuroinflammation by upregulating several pro‐inflammatory factors.^[^ ^28^ ^]^ Therefore, 14 days post‐TBI injury, immunostaining of Iba1 (to label microglia and central nervous system (CNS) infiltrating monocytes/macrophages) and GFAP (to label astrocytes) was conducted. **Figure** **3**A,B were the representative images of GFAP and Iba1 labeling in the cortex and hippocampus of TBI rat brain. The number of endpoints around the microglia was dramatically reduced in the TBI, TBI+ BT, and TBI+ bone transport control groups (TBI+BT group) compared to the Sham group, and the TBI group had a significantly higher percent area of GFAP and Iba1 coverage. Moreover, astrocytes showed that cell hypertrophy and synaptic elongation in the TBI group. There was an overall decrease in Iba1 level in the TBI+BT group compared to the TBI group in the cortex region and hippocampus, no significant reduction of GFAP level in the TBI+BT group compared to the TBI group (Figure 3C‐F). To further investigate microglia activation, the morphology of microglia was analyzed by Sholl analysis, each data point represents the number of Iba1 + branches intersecting with a radius of 0–60 µm from the microglia soma in Figure 3G, and the results showed significant differences between TBI and TBI+BT groups from 30–40µm. In sham group, microglia characterized by long protuberance and small soma. The reactive microglia presented retracted protuberance and a larger, rounder soma in TBI group; the microglia activation was alleviated in BT group by showing more endpoints compared to TBI group (Figure 3H). These results suggest that the neuroinflammation in the ipsilateral cortex and hippocampus was reduced in the TBI rats treated following CBT treatment.

### 2.4 Ablation of Meningeal Lymphatic Vessels Reversed the Therapeutic Effects of CBT

**Figure 4:**
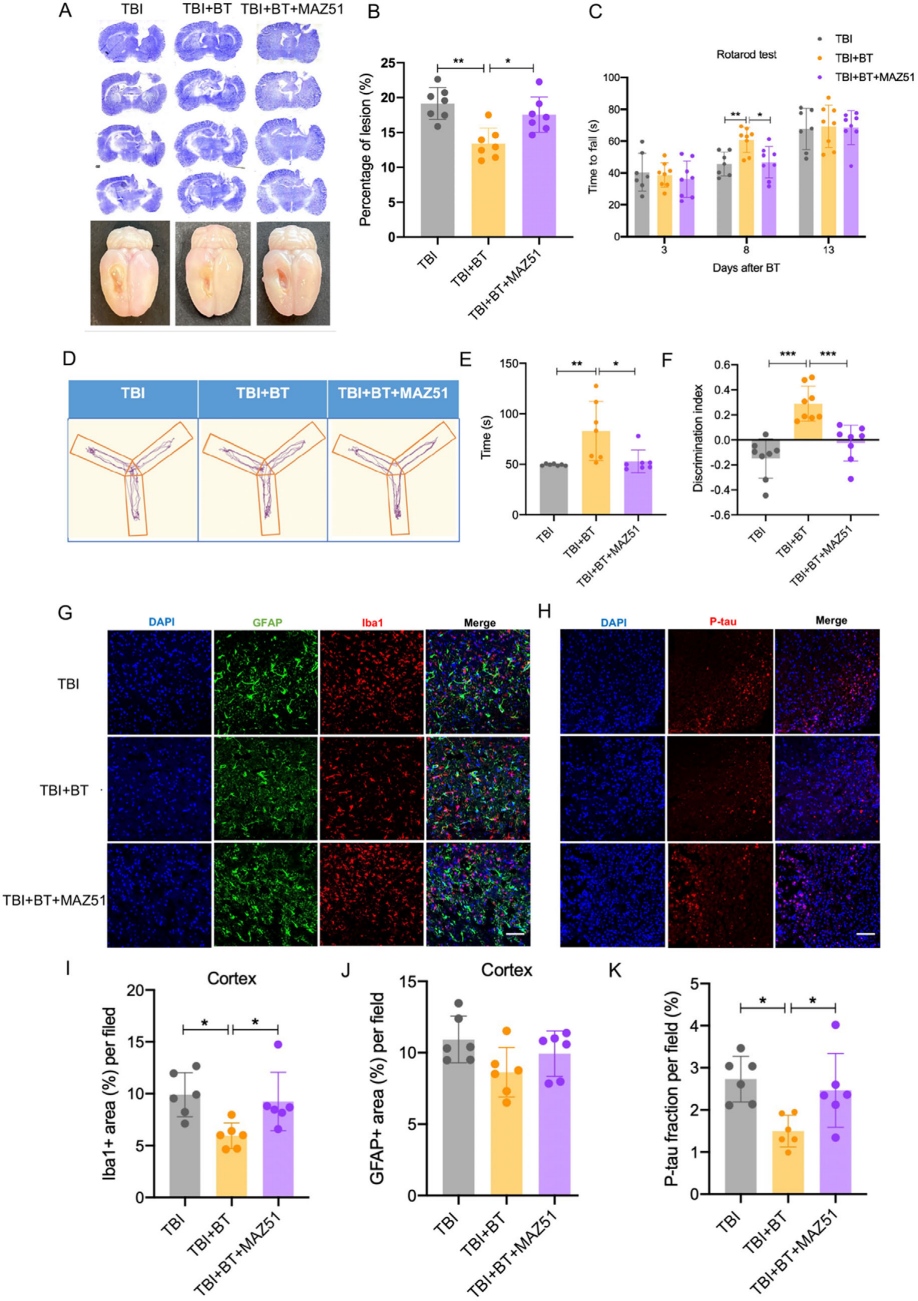
Ablation of meningeal lymphatic vessels reversed the therapeutic effects of CBT. A) Representative Nissl staining and images of gross view of the TBI rats injected with MAZ51. B). Quantification of percentage of lesion size, calculated by ratio of the lesion area in ipsilateral brain to whole area of contralateral brain. C) Time to fall from the rotating rod of TBI rats significantly increased after 3, 8, and 13 days of BT surgery. D) Representative images of Y maze track in three groups. E) Duration of rat staying in novel arms of Y maze in three groups. F) Histograms represent the discrimination index (DI) for each experimental group. Discrimination index = (time spent with novel object − time spent with old object)/total exploration time. G) Representative images of perilesional cortex stained with GFAP (green) and Iba1 (red), scale bar = 50 µm. H) Representative images of perilesional cortex stained with P‐tau, scale bar = 50 µm. I) Quantification of Iba1 fraction in three groups. J) Quantification of GFAP fraction in three groups. K) Quantification of P‐tau fraction in three groups. ^*^ *p* < 0.05, ^**^ *p* < 0.01, ^***^ *p* <0.001. Data are shown as mean ± SD.

To better understand the meningeal lymphatic function modulation during CBT treatment in TBI rats, we next explored whether ablation central nervous system (CNS) lymphatic drainage effectively reverses CBT effects. To this end, we utilized delivery of MAZ51 into Cerebrospinal Fluid (CSF), a VEGFR3 kinase inhibitor, which has previously been shown to effectively block the growth and drainage function of the MLVs.^[^ ^29^, ^30^ ^]^ Firstly, we examined the ablation effects of MAZ51 in MLVs coverage and function of SD rats. MAZ51 in dimethyl sulfoxide was dissolved in 20% SBE‐β‐CD and artificial CSF, then loaded into a capsule of the osmotic pump with a total volume of 100 µL, continuously intra‐cisterna magna infusion at 1mg/kg of body weight for 30 days. Not surprisingly, MLVs underwent regression after treatment with MAZ51, the LYVE‐1 positive area in sagittal sinus and middle meningeal artery region (MMA) were both reduced compared to control group injected with dimethyl sulfoxide (Figure S2A,C,D, Supporting Information). The quantification of OVA‐A647 tracer in dCLNs was decreased (Figure S2B,E, Supporting Information), suggesting MAZ51 reduced MLVs coverage and ablated meningeal lymphatic draining function. Although VEGF‐C primarily stimulates lymphangiogenesis by activating VEGFR‐3, it can also act on blood vessels under certain conditions. To determine whether MAZ51 will affect cerebral vessels, we performed CD 31 IF staining and found that cerebral vessel coverage showed no significant difference between MAZ51 group and control group in cortex, striatum and hippocampus region. (Figure S3A‐F, Supporting Information). In addition, MAZ51 has no detrimental or favorable effects on cognitive, memory and motor function of rats, reflected by no significant differences between control group and MAZ51 group in the results of novel objection test, Y maze and catwalk gait analysis (Figure S4A‐M, Supporting Information). Notably, the TBI rats underwent CBT who experienced MAZ51 injection had a significantly larger lesion and a higher fraction of lesion size (**Figure** **4**A,B) compared to TBI+BT group. Rats in TBI+BT+MAZ51 group also performed significantly worse on the rotarod tests, Y maze test and novel objection test, as revealed by reducing duration on the rod and novel arms of Y maze (Figure 4C‐E). The discrimination index in the NOR test showed significant differences between the TBI+BT+MAZ51 and TBI +BT groups (Figure 4F). To further confirm that inhibition of meningeal lymphatic function may reverse anti‐neuroinflammation of CBT treatment, we used GFAP and Iba1 to label astrocytes and microglia respectively. Evidently, compared with TBI+BT group, the expression of Iba1 reduced significantly in TBI+BT+MAZ51 group (Figure 4G,I,J). MAZ51 also eliminated the CBT improved effect on P‐tau clearance, as reflected by a robust increase P‐tau aggregation in ipsilateral cortex (Figure 4H,K). These data indicated that meningeal lymphatic function ablation in TBI rats would be effective in limiting CBT therapeutic effects.

### 2.5 CBT Enhanced Bone Healing in TBI Rat Skull Defects

**Figure 5:**
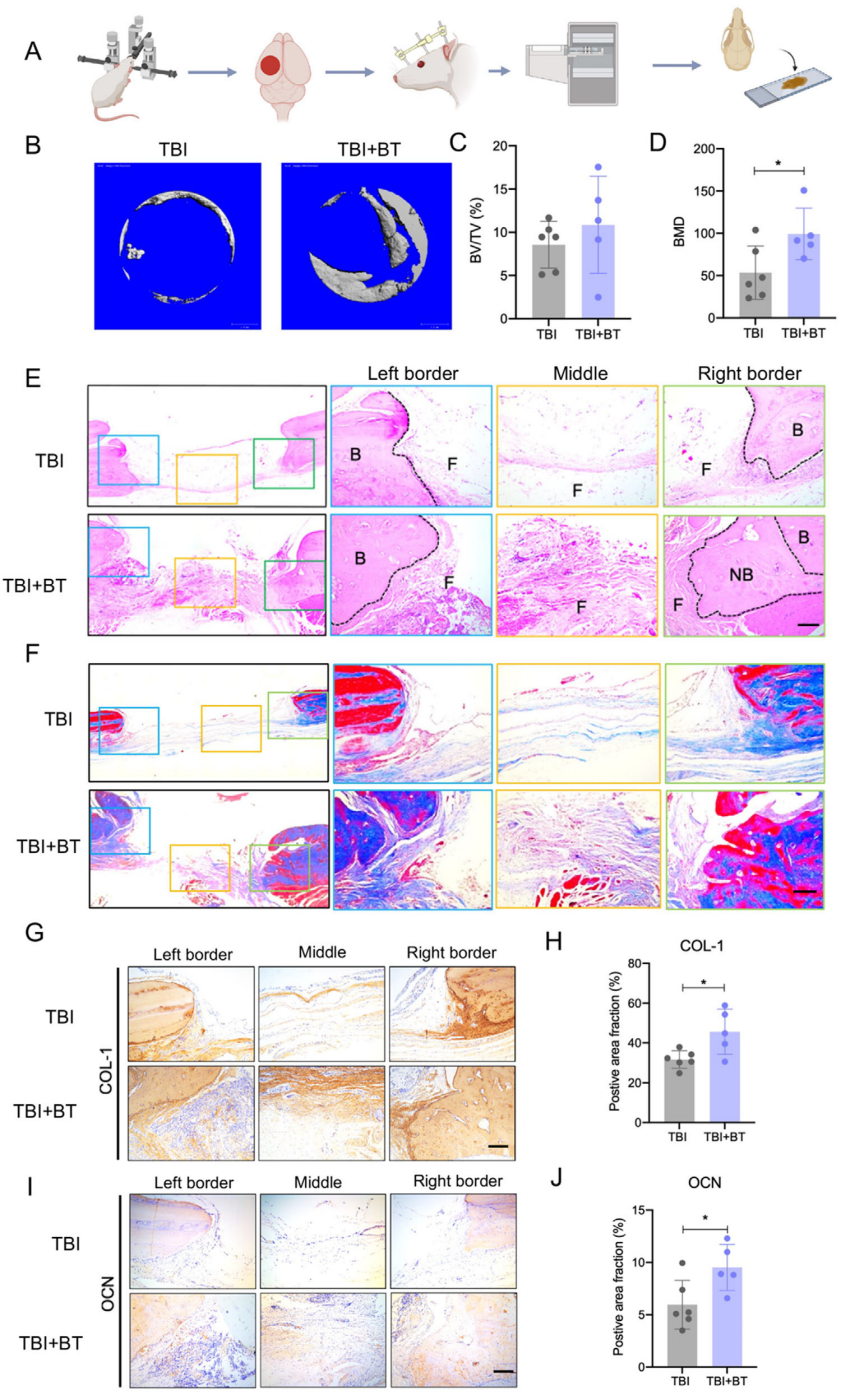
CBT enhanced bone healing and promoted osteogenic marker expression in TBI skull defects. A) Schematic of the experimental layout. CBT surgery was performed 24 h after the establishment of TBI model, after BT treatment for 13 days, and a bone consolidation period for further 2 months. Skull samples were collected for micro‐CT and histology analysis. B) Representative images of micro‐CT reconstruction in the two groups. Scale bar = 1 mm. C) Quantitative bone volume analysis (BV: bone volume; TV: total defect volume). D) Quantitative bone mineral density measurement. (E) Representative images of H&E staining in the two groups, scale bar = 100 µm. B: border of bone defect; NB: new bone, F: fibrous tissue. F) Representative images of Masson staining in the two groups, scale bar = 100 µm. G,I) Representative images of COL‐1 and OCN immunohistochemical staining of the skull, scale bar = 100 µm, H) Quantification of COL‐1 positive area of bone defects region in two groups. J) Quantification of OCN positive area of bone defects region in two groups. ^*^ *p* < 0.05, ^**^ *p* < 0.01, ^***^ *p* < 0.001. Data are shown as mean ± SD.

To assess the bone formation following CBT in TBI rats, we collected the skull samples 2 months after TBI treatment (**Figure** **5**A). Micro‐CT reconstruction data showed substantially more new bone formation in the TBI skull defects treated by CBT compared to the TBI group without CBT treatment (Figure 5B). Although quantification of Bone volume/tissue volume (BV/TV) results did not show any differences between the two groups, the bone mineral density (BMD) in the CBT group increased significantly (Figure 5C,D). To further confirm the effects of CBT on the repair of skull bone defect, the skull sections were subjected to Masson's trichrome staining and HE staining. Figure 5E showed CBT resulted in a slightly increased newly formed bone formation compared with the TBI group. The images in Figure 5F showed an increase in the newly formed connective tissues, which were stained blue color with Masson's trichrome staining. Furthermore, the immunohistochemical staining against type I collagen (COL‐1) and osteocalcin (OCN) on the histological sections revealed elevated bone matrix deposition and increased expression of osteogenic markers in the TBI +BT group as compared to the TBI group (Figure 5G–J).

### 2.6 CBT Showed Long Term Beneficial Effects on Brain Injury and Neuronal Death in TBI Rats

**Figure 6:**
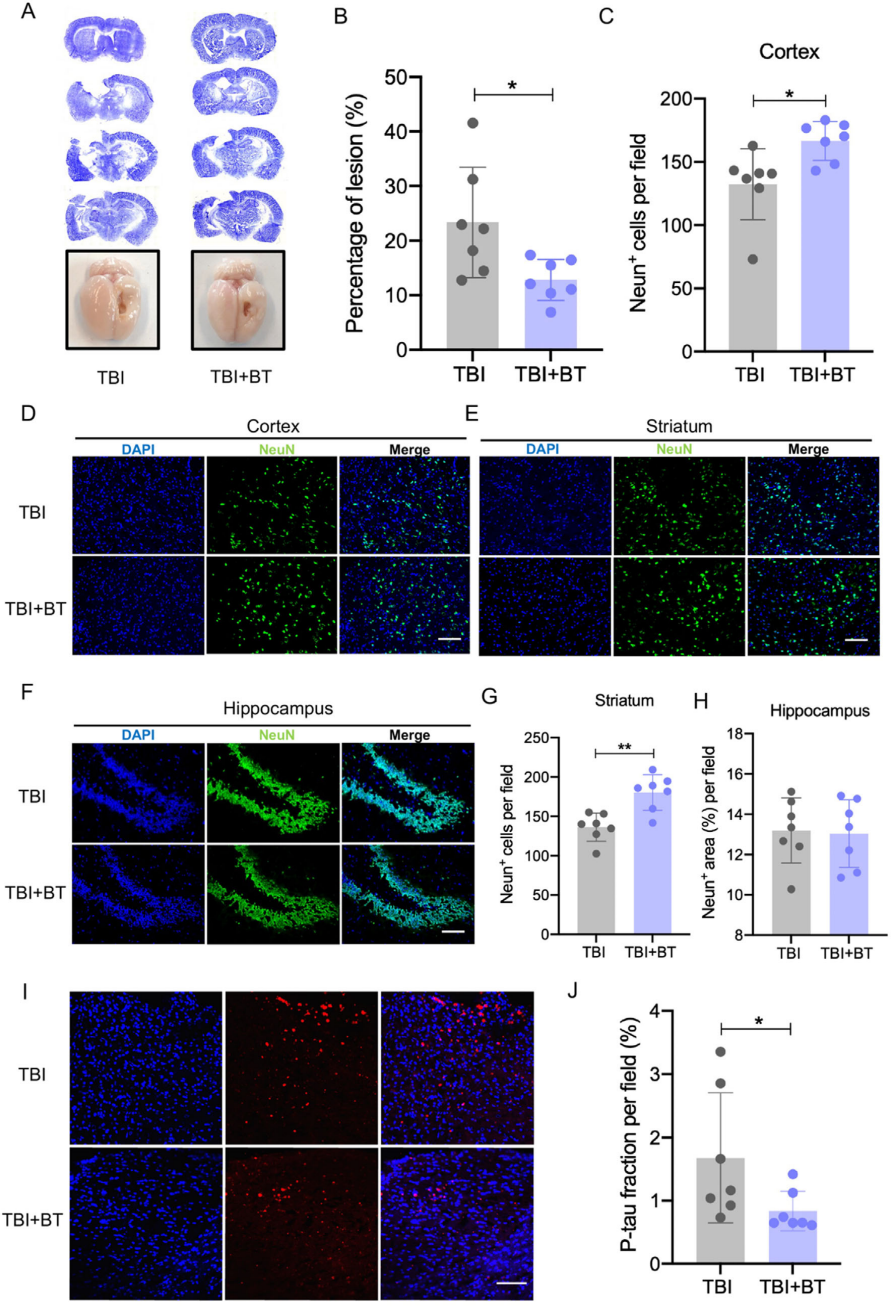
CBT treatment reduced lesion size and increased the neuronal cells survival in the perilesional cortex 2 months post‐TBI. A) Representative Nissl staining and images of gross view of TBI rats treated with CBT after 2 months. B) Quantification of percentage of lesion size, calculated by ratio of the lesion area in ipsilateral brain to whole area of the contralateral brain. C) Quantification of total viable neurons in the perilesional cortex. D) Representative image of the perilesional cortex section showing NeuN+ immunofluorescence at 2 months after TBI, scale bar = 100 µm. E) Representative image of the striatum section showing NeuN+ immunofluorescence at 2 months after TBI, scale bar = 100 µm. F) Representative image of the hippocampus section showing NeuN+ immunofluorescence at 2 months after TBI, scale bar = 100 µm G) Quantification of total viable neurons in the striatum. H) Quantification of total viable neurons in the hippocampus. I) Representative images of perilesional cortex stained with P‐tau (red) and DAPI (blue), scale bar = 50 µm. J) Quantification of P‐tau fraction in two groups. ^*^ *p* < 0.05, ^**^ *p* < 0.01, ^***^ *p* < 0.001. Data are shown as mean ± SD.

Next, we evaluated whether CBT exerts long‐term improved neurological function in TBI rats, the brain samples were collected 2 months after the CBT treatment. The Nissl staining results showed that the lesion area in the TBI+BT group was slightly reduced compared to the TBI group, and the gross view also displayed a decreased lesion area 2 months after TBI induction (**Figure** **6**A,B). We further quantified the number of neurons in cortex, striatum and hippocampus region. Despite there being no significant differences between the TBI and TBI+BT group in the hippocampus, the number of NeuN+ was significantly increased in the cortex and striatum region 2 months after CBT treatment (Figure 6C‐H), indicating that CBT has long‐term beneficial effects in neurological function recovery in TBI rats. Long‐term of P‐tau deposition in TBI may induce neurodegeneration pathology, so we performed P‐tau staining and found its aggregation in the cortex was still reduced after CBT treatment for 2 months (Figure 6I,J), indicating that CBT may alleviate the risk of neurodegenerative disorders in TBI rats.

### 2.7 CBT Improves Neurological Function in TBI Patients with Decompressive Craniectomy

**Figure 7:**
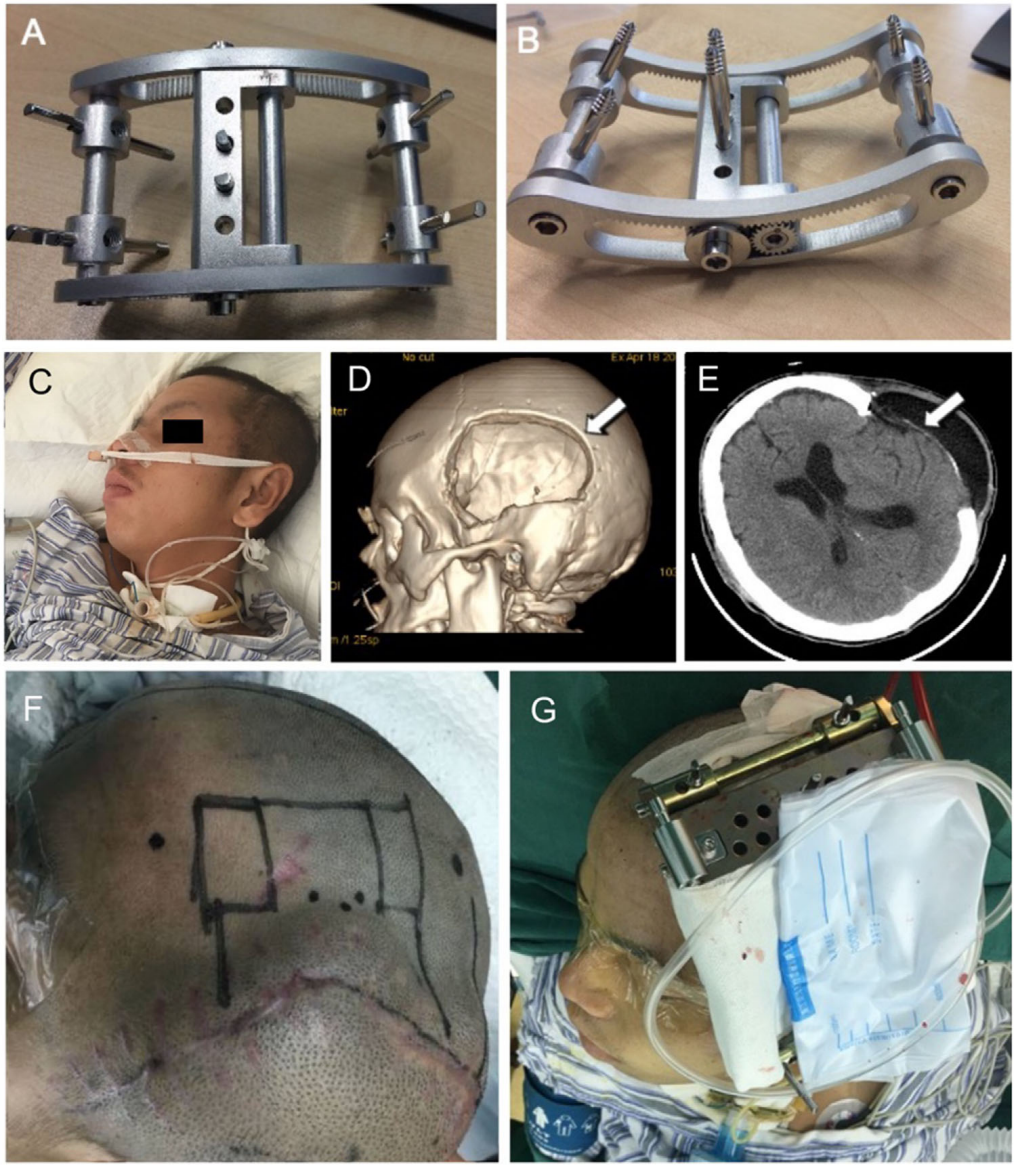
An 18‐year man suffered TBI was performed CBT surgery. (A‐B) Structure of the external fixator: anterior view A) and dorsal view B). C) Patient was in a state of mild disturbance of consciousness with a trachea intubation to maintain ventilation. D) CT reconstruction showed a bone defect in the left skull after decompressive craniectomy for about 4 months. E) Representative CT Image of horizontal position indicated large defects in parietal region. F) A bone flap was designed to be obtained in the rim of bone gap border. G) View of patient with the external fixator after surgery.

In order to further investigate the therapeutic effects of CBT on TBI patients, we redesigned and revised the structure of cranial external fixation device to make it simpler and easier to handle (**Figure** **7**A,B), CBT surgery was performed in two individuals more than three months after decompressive craniectomy, all the patients gave informed consent. The results showed the neurobehavioral disorder was dramatically improved after surgery in two patients.

Case 1: An 18‐year‐old young adult sustained a closed head injury from a car accident. After decompressive craniectomy for about 4 months, he was still in a state of mild disturbance of consciousness with trachea intubation to maintain ventilation (Figure 7C). His eyes were dim, and he was indifference to himself and his surroundings with short and slow response, and a lack of orientation to person, place, time, or circumstances. Patients' arm and legs are difficult to walk, stretch, stand, and sit upright because of muscle weakness. The CT results revealed there was also a large area of skull defect in the left temporal bone and part of parietal bone after craniectomy (Figure 7D,E).

Then we planned to perform CBT surgery for him. Patient underwent general anesthesia after estimating and designing the incision area, direction, and bone graft region. We chose the frontal, parietal tubercles, and the sagittal suture as three external landmarks during the operation, then designed a bone flap from the right parietal bone, which is the border of the bone gap (Figure 7F). After dissection of the skin and periosteum about 2 cm, holes are established in the left side of skull to allow pins to be placed later. Then, a bone graft 5 cm×5 cm was obtained just next to the bone defect region and tried to keep the periosteum intact. The bone flap was placed along right border of the bone defect. After fixing the external fixator and tightening all the screws, the incision was closed, and a drainage was placed (Figure 7G). After five latency days, bone transport was started with a speed of 1mm per day, performed twice a day. The period of bone transport depended on the distance between bone flap and the opposite rim of bone defect, it was terminated once it reached the rim. Then the bone flap was fixed on the frame. After 3 months of fixation, the external frame was removed under anesthesia.

**Figure 8:**
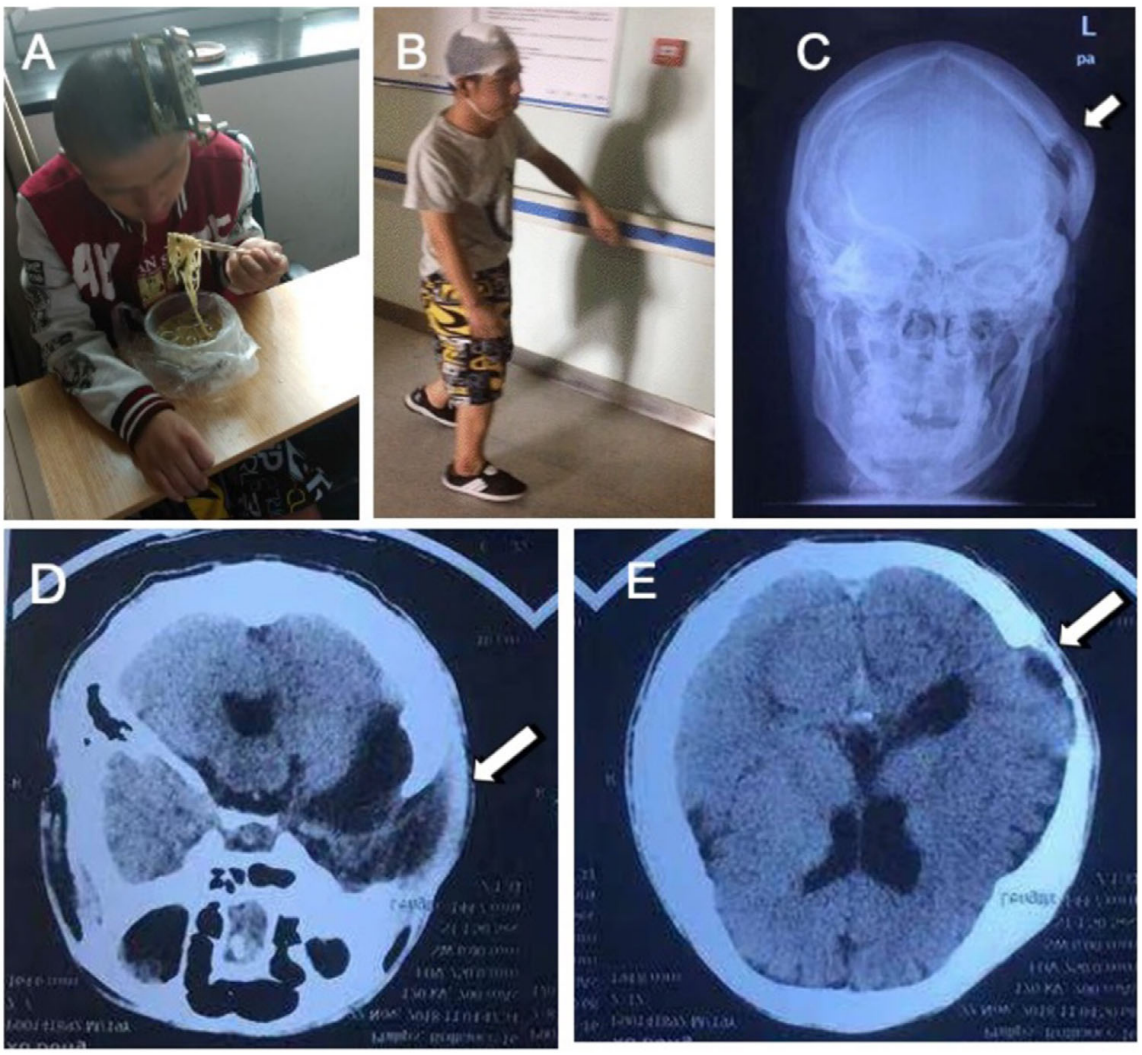
Therapeutic effects of CBT on case 1. A) Patient was able to feed himself 2 months after operation. B) He can walk slowly after removal of the external device. C) X‐ray results suggested bone transport repaired the bone gap. D) Representative CT Image of coronal position showed defects in temporal region were reduced. E) Representative CT Image of horizontal position showed bone defects were repaired.

Two months after the operation, the patient was able to feed himself (**Figure** **8**A). His mental state was more active compared to pre‐operation, and he can talk to people initiatively. After removal of the fixator, the patient can walk by himself, whereas he got around with other support several months ago (Figure 8B). The X‐ray showed the cranial defect was successfully repaired by the CBT surgery (Figure 8C). CT Images of the horizontal position and coronal position displayed that the size of defect was reduced (Figure 8D,E).

## 3 Discussion

The present study introduced a novel and simple surgical intervention CBT, could significantly improve the outcomes of brain recovery in TBI rats. The meningeal lymphatic drainage function and P‐ tau clearance were all enhanced while Iba1‐induced neuroinflammation was migrated following CBT treatment. Moreover, ablation of meningeal lymphatic function reversed the therapeutic effects of CBT in TBI rats, indicating, maintaining meningeal lymphatic function is indispensable for the effect of CBT on TBI neurological recovery. Most importantly, CBT treatment accelerated skull defect healing in TBI rats and showed long‐term beneficial effects on neurological function recovery.

The bone transport based on the Illizarov technique has been applied in tissue regeneration, which is not limited to bone repair.^[^ ^19^, ^31^ ^]^ It was found that the number of muscle fibers was increased with the duration of distraction and the muscle volume in patient who underwent mandibular lengthening was increased significantly.^[^ ^32^ ^]^ Moreover, the stimulation of deep fascia regeneration was demonstrated by increased collagen type III in the distraction group.^[^ ^33^ ^]^ In addition, regeneration of blood vessels is critical for bone healing during bone transport, it is reported that the number of new blood vessels is most abundant in the early period of distraction but tends to be a more mature histological structure in the late period.^[^ ^34^ ^]^ Meanwhile, transverse bone transport has been reported to facilitate ulcer healing in diabetic foot.^[^ ^35^ ^]^ These findings proved that gradual distraction contributes to regenerating tissue and maintaining cell proliferation, which is also the core of the Ilizarov technique. Based on the above evidence, it is likely that the gradual tension stimulation may stimulate tissue regeneration and then further enhance brain recovery. Furthermore, we have demonstrated that CBT promotes brain functional recovery in MCAO rats by enhancing angiogenesis, neurogenesis and meningeal lymphatic function in previous publications.^[^ ^20^ ^]^ As TBI shares a similar pathology with ischemic stroke, therefore we proposed that application of CBT may also be beneficial for TBI brain injury. CBT procedure transports the cranial bone flap which stimulates neuronal recovery and at the same time the bone transport could also facilitate cranial reconstruction after decompressive craniectomy.

The MLVs are widely thought to be responsible for draining macromolecules, immune cells, and excess fluid from the CNS to dCLNs, then further circulate to peripheral lymphatic system.^[^ ^36^, ^37^ ^]^ As a pivotal role in the exchange between CSF and the interstitial fluid, the participation of MLVs has been identified by mounting studies.^[^ ^38^, ^39^ ^]^ Previous studies have shown that MLVs dysfunction aggravates TBI neurological deficits and neuroinflammation and TBI leads to impairments in meningeal lymphatic drainage and improving the function of MLVs contributes to promoting brain edema absorption after TBI.^[^ ^40^ ^]^ Additionally, P‐tau accumulation in the brain is detrimental for TBI outcome and has been proposed as a diagnostic and prognostic biomarker for acute TBI.^[^ ^41^ ^]^ P‐tau aggregation was exacerbated when the lymphatic system was impaired in the AQP4 mice,^[^ ^8^ ^]^ indicating that malfunction of meningeal lymphatic may correlate with P‐tau accumulation in TBI conditions. Moreover, malfunction of MLVs exacerbated neuroinflammation in TBI conditions and augmentation of meningeal lymphatic drainage function rescued gliosis in TBI.^[^ ^7^ ^]^ It is found that blocking meningeal lymphatic function led to release of IL‐1β, IL‐6, and TNF‐α and active gliosis in mouse brain.^[^ ^42^ ^]^ Improvement of meningeal lymphatic function ameliorated neuroinflammation in cirrhotic rats by reducing expression of IL‐1β, IFN‐γ, TNF‐α, and Iba1.^[^ ^43^ ^]^ Additionally, microglial neuroinflammation contributes to tau accumulation in chronic traumatic encephalopathy^[^ ^44^ ^]^ and P‐tau oligomers bind to microglia and perpetuate neuroinflammation.^[^ ^45^ ^]^ Therefore, neuroinflammation and p‐tau aggregation form a destructive feedback loop, and both were regulated by MLVs function. Our findings showed CBT treatment significantly enhanced the meningeal lymphatic function in TBI rats, decreased microglia but no significant changes in astrocyte numbers were observed after CBT treatment. Compared to microglia, astrocytes and the meningeal lymphatics interact indirectly, astrocytes are more responsive to parenchymal damage than meningeal lymphatic changes.^[^ ^46^ ^]^ Meningeal lymphatics primarily influence peripheral immune cells and microglia, but minimal impact on astrocyte‐activating pathways,^[^ ^47^ ^]^ and studies have showed VEGF‐C treatment reduced microglial Iba1+ staining but no change in GFAP+ astrocytes in TBI model.^[^ ^7^ ^]^ This might explain why astrocyte infiltration did not improve after CBT treatment, and inflammatory cytokine levels should be measured to further confirm CBT effects on neuroinflammation. Collectively, these findings suggest that targeting MLVs and lymphatic function may provide a new strategy for accelerating P‐tau clearance and alleviating neuroinflammation to alleviate brain injury in TBI.

VEGFC/VEGFR3 has been demonstrated to be a key pathway to regulate lymphatic endothelial cell proliferation, migration, and maintain lymphangiogenesis,^[^ ^48^ ^]^ VEGFR inhibitor (MAZ51) selectively inhibits VEGFR‐3 tyrosine kinase activity, blocking downstream signaling pathways that promote lymphatic endothelial cell proliferation and migration. We found that the CBT treatment effects were diminished after MAZ51 administration, indicating VEGFC/VEGFR3 pathway is indispensable for CBT to exert its effects. It was demonstrated that expression of VEGFR3 was elevated gradually started from distraction period,^[^ ^49^ ^]^ and expression of VEGFC was proved to increase in latency, distraction, and consolidation phase.^[^ ^50^ ^]^ VEGFD was also detected during latency and distraction period, which activates VEGF receptors and promotes growth and remodeling of lymphatic vessels.^[^ ^50^, ^51^ ^]^ Although there was no obvious lymphangiogenesis after CBT in TBI rats, the enhanced meningeal lymphatic drainage function may contribute to improved lymphatic endothelial function stimulated by VEGFC/VEGFR3 pathway released during bone transport. Additionally, the OVA tracer trafficking in dCLNs indicates the output function of meningeal drainage, that means how effectively they transport CSF/ interstitial fluid and solutes from the brain to peripheral lymphatics. The OVA tracer collected in MLVs reflects its permeability and structural patency.^[^ ^38^ ^]^ There is a slight increase in OVA in MLVs but more notable augment in dCLNs, indicating the CBT treatment has a more significant effect on output function of meningeal drainage and a weaker effect on lymphatic permeability and structural patency. Moreover, it was reported that during bone distraction osteogenesis, anti‐inflammatory cytokines such as IL‐10 and TGF‐β were released systemically, and the macrophages at the bone formation site were also polarized from pro‐inflammatory M1 type to pro‐repair M2 type.^[^ ^52^ ^]^ These factors may also mitigate the neuroinflammation in the brain of TBI rats, which needs further investigation.

Decompressive craniectomy is performed widely for the treatment of severe traumatic brain injury, is a surgical procedure to relieve intracranial hypertension and brain swelling, involving removing a piece of cranial bone segment.^[^ ^53^ ^]^ CBT has since been used in cranial defects reconstruction and correction for craniofacial abnormalities, therefore, it may be an ideal choice for cranial bone repair for patients undergone decompressive craniectomy.^[^ ^54^, ^55^ ^]^ CBT is achieved by gradually moving an autologous bone graft, which has been widely used as the golden standard in cranioplasty in contrast to other synthetic biomaterials.^[^ ^56^, ^57^ ^]^ Clinical studies showed that autologous bone grafts lead to excellent cosmetic results, 86.2% of patients were satisfied with the treatment outcome.^[^ ^58^ ^]^ Autologous bone graft has excellent biocompatibility, low risk of infection, and non‐union.^[^ ^59^, ^60^ ^]^ In addition, autologous bone grafts have good osteoconductive potentials, the osteointegration at the defect side further promotes vascularization, cell migration, and new bone regeneration.^[^ ^61^ ^]^ By slowly transporting a bone segment adjacent to the defect border, CBT contributes to new bone formation after bone consolidation period. The mechanical tension applied to the bone segment and gap affects the cellular activity profoundly.^[^ ^62^ ^]^ It has been proposed that focal adhesion kinase (FAK)‐mitogen‐activated protein kinase (MAPK), Hippo, and wingless‐related integration site (Wnt) signaling pathway expression were upregulated directly after mechanical stimulation during DO by microarray technology.^[^ ^63^, ^64^ ^]^ The transport mechanical force then further activates various growth factors and cytokines such as BMP2, BMP4, and TGF‐β that promote bone formation and remodeling.^[^ ^65^, ^66^ ^]^ Moreover, the accelerated fracture healing in the presence of traumatic brain injury has been proved in both preclinical and clinical studies, so it is possible that the skull bony flap healing also accelerates under the condition of brain injury.^[^ ^67^ ^]^ Therefore, this evidence shows CBT may be a new alternative method for repairing larger cranial defects.

There are some limitations in this study. First, the CBT surgery protocol is based on previous studies and experiences in distraction osteogenesis treatment of long bones. The latency period, bone transport rate, rhythm, and bone consolidation period all determine bone formation.^[^ ^68^, ^69^ ^]^ Thus, different parameters should be compared and evaluated to determine the optimal CBT treatment regimens for cranial bone formation. Second, although MAZ51 is able to block the proliferation of VEGFR3‐expressing endothelial cells and has been used to induce MLVs regression in lots of studies, it inhibits multiple signaling pathways, slows proliferation and induces apoptosis of various cells that do not express VEGFR‐3,^[^ ^70^, ^71^ ^]^ so alternative approaches soluble VEGFR‐3 (VEGF‐C trap) or rat model with lymphatic Vegfr3‐deletion should be used to provide more convincing data on the specific effect of blocking VEGFR‐3. Third, a moderate controlled cortical impact TBI rat model was used in this study, the neurological deficits recover faster compared to patients with TBI, especially motor function. Therefore, severe controlled cortical impact TBI model or other types of TBI animal models should be applied to evaluate long term neurological deficits in future studies.^[^ ^72^ ^]^ Fourth, although the CBT surgery is a minimal invasive surgery, there are still potential risks should be considered when preforming clinically, such as the free bone segment may be loose after CBT surgery, and reoperation is required to fix it if necessary. Scalp and skull infection may occur after surgery and lead to intracranial infection and nonunion. Therefore, neurosurgeons should be careful to avoid the above risks when conducting CBT surgery. Lastly, we proved MLVs are a crucial mediator for CBT treatment in TBI, it is necessary to find the key molecular pathways that govern these effects. Exploring the underlying mechanisms of CBT therapy in TBI conditions will shed light on future clinical applications of CBT in the management of other neurological disorders.

Taken together, this current study investigates the therapeutic effects of CBT in TBI, and demonstrates that CBT enhances meningeal lymphatic function, reduces neuroinflammation, and increases P‐tau clearance, the CBT treatment effects were reversed after VEGFC pathway ablation. CBT treatment accelerated skull defect bone repair in addition to its promoting effects on neurological function recovery, indicating that CBT treatment is a promising new therapeutic strategy for TBI clinical management. Whether the CBT can benefit TBI patients and improve brain outcomes warrants further investigation.

## 4 Experimental Section

### Animals

Sprague‐Dawley (SD) rats (8‐10 weeks old, male) were obtained from the Laboratory Animal Research Centre of the Chinese University of Hong Kong. All rats were housed under a 12 h light/dark cycle with controlled room temperature (23 ± 1°C) and unlimited access to food and water. All surgeries were performed under anesthesia, and efforts were made to minimize the suffering of the animals. All animal experiments were carried out in Li Ka Shing Institute of Health Sciences, Prince of Wales Hospital, with the approval of the Department of Health of the Government of Hong Kong and the Chinese University of Hong Kong Animal Experimentation Ethics Committee (No. 19‐211‐MIS).

### TBI Model

The controlled cortical impact model (CCI) was used in this study as previously described.^[^ ^73^ ^]^ SD rats were anesthetized with ketamine (80–100 mg kg^−1^) and xylazine (10 mg kg^−1^) in saline, then placed in a prone position with the head skin shaved and sterilized, a toe pinch test was used to verify if the rats were anesthetized properly. A midline incision (≈2 cm) was made to expose the skull. A U‐Frame stereotaxic instrument was used to fix the heads of rats. The ear bars were placed into the ear canal and the incisor was placed over the incisor bar. A skin retractor was used to fully expose the skull. The muscle and fascia were then stripped from the skull. A full‐thickness bone defect (diameter 5 mm) was made at the left calvarial region using a circular low‐speed saw carefully to avoid damaging any brain tissues. The location of craniectomy was between the sagittal suture and coronal suture which is nearly the bregma. Saline was dripped during craniectomy to lower the temperature. The speed of the impactor device was set at 5 m s^−1^ with an impact duration of 500 ms. The tip of impact lightly touched the exposed dura. A Vernier caliper was used to set the depth of impact as 3 mm, then the injury was induced by an impactor with a diameter of 3 mm. The skin incision was then closed with 4‐0 nylon sutures. Finally, the rat was removed from the stereotaxic frame and put on a heating pad during the recovery from anesthesia. For the sham control group rats, only craniectomy was conducted.

### CBT Surgery

The detailed procedure of CBT surgery was described in our previous study.^[^ ^20^ ^]^ A custom‐made external transport device was used for CBT procedures in SD rats (Figure 1A, Jinlei Technology Co. Ltd, Tianjin, China). Twenty‐four hours after the establishment of the TBI model, A bone flap with a diameter of 5 mm was made next to the calvarial defect region after TBI establishment using a circular low‐speed saw (5 mm in diameter) with careful protection of dura. Four holes around the bone defect were drilled on the skull for screws to attach the external fixator device, and 1 screw penetrating the bone flap was attached to the device's sliding slot for bone transport (BT) in the following days. The skin incision was closed with 4‐0 nylon sutures. After a latency of 3 days, the bone flap transport was initiated with a rate of 0.25 mm per 12 h and lasted for 10 days, by adjusting the sliding slot screw on the device. For the TBI+BTC group, rats received craniectomy which was fixed with the same external device, but the bone transport procedure was not applied. The CBT surgery took ≈15 min to complete with a 95% success rate. X‐ray was used to check the location of the cranial bone flap and fixator position after surgery, about 5% of rats who died of anesthesia or had fixator loosening or inappropriate fixation or position during surgery, were terminated immediately. Buprenorphine HCI (0.05 mg kg^−1^) was administrated 30 min before surgery and a heating pad was provided after surgery to maintain body temperature during surgery and recovery. Buprenorphine HCI (0.05 mg kg^−1^) was given by intraperitoneal injection twice daily for five consecutive days after surgery for pain and stress relief. Body temperature, body weight, food, and water intake, and the conditions of surgical incision were checked daily for the whole experimental duration.

### OVA‐A647 Cisterna Magna Injection

SD rats were anesthetized, and an incision (≈1 cm) was made. A micro‐syringe with a 32G needle was used to inject 50 µL OVA‐A647 (O34784, Thermo Fisher Scientific, USA) at 0.5 mg mL^−1^ in artificial CSF (597316, Harvard Apparatus, UK) into cisterna magna with a rate of 2.5 uL min^−1^. The syringe was left in place for an additional 10 min to prevent backflow of CSF after injection.

### MAZ51 Administration

MAZ51 in dimethyl sulfoxide (Cat. No. HY‐116624, MedChemExpress, USA) was dissolved in 20% SBE‐β‐CD (Sulfobutylether‐β‐Cyclodextrin, Cat. No. HY‐17031, MedChemExpress, USA) and artificial CSF (597316, Harvard Apparatus, UK), then loaded into a capsule of osmotic pump (1004W, RWD life sciences, Shenzhen, China) with total volume of 100 µL, continuously intra‐cisterna magna infusion at 1mg kg^−1^ of body weight for 30 days. The osmotic pumps were connected to a 33G needle with a limited length of 1.5 mm and incubated overnight in a 37 °C water bath. The tiny needle of osmotic pump was inserted into and fixed at the cisterna magna, while the pump was placed subcutaneously, which can achieve continuous intra‐cisterna magna infusion at a rate of 0.125 µL h^−1^ for 4 weeks. The control group was given the same volume of vehicles. On the 30th day, TBI model was established and followed by CBT surgery. On the 14th day after TBI induction, animals were killed for analysis.

### Immunofluorescence Staining

Rats were given a lethal dose of anesthetics by i.p. injection of 20% pentobarbital and were then transcardially perfused with ice‐cold PBS. The harvested brain and dCLNs were fixed in 4% paraformaldehyde (PFA) overnight at 4 °C, and were dehydrated using 10%, 20%, and 30% (w/v) sucrose solution subsequently. Samples were embedded in OCT (optimal cutting temperature) compound (4583, Sakura Finetek, USA), and 10‐µm or 30‐µm or 60‐µm sections were acquired using a freezing microtome (NX70, Thermo Fisher Scientific, USA).

For immunofluorescence staining, fresh‐frozen brain and dCLNs sections were fixed with 4% PFA for 15 min, rinsed, and washed in phosphate buffer saline (PBS) for 5 min and 3 times. Sections were incubated in 0.5% Triton X‐100 for 15 min and blocked using 2% donkey/goat serum and 1% bovine serum albumin (BSA, SRE0098, Sigma‐Aldrich, USA) for 1 h at room temperature. This blocking step was followed by incubation with appropriate dilutions of primary antibodies: anti‐CD31 (AF3628, R & D System, 10 ug mL^−1^), anti‐GFAP (ab7260, Abcam, 1:300), anti‐Iba1 (ab5076, Abcam, 1:300), anti‐NeuN (ab177487, Abcam, 1:300), anti‐P‐tau (MN1020, Abcam, 1:200) overnight at 4 °C. Sections was washed in PBS and followed by incubation for 1 h at room temperature in dark with secondary antibodies: donkey anti‐rabbit Alexa Flour 488 (ab150073, Abcam, 1:500), donkey anti‐goat Alexa Flour 555 (A‐21432, Invitrogen, 1:500), and donkey anti‐mouse Alexa Flour 594 (R37115, Invitrogen, 1:500) diluted in PBS with 1% (w/v) BSA and 2% (v/v) of serum (donkey). 4′,6‐diamidino‐2‐phenylindole (DAPI) was used to label cell nucleus and then samples were mounted with proLong™ Gold Antifade Mountant (P36934, Thermo Fisher Scientific).

### Whole Mount Staining

Before whole mount staining, the skin and muscle tissues were carefully dissected away, and the skull with meninges was fixed in 4% PFA for 24 h at 4 °C. Put the skull in a 10 cm cell culture dish. Under the dissecting microscope, gently detach the meninges from the skullcap along the sagittal and transverse sinuses, and then remove the entire meninges using a fine forceps, small pieces of skull bone attached to the meninges should also be removed. The meninges were permeabilized with 0.5% Triton X‐100 and incubated with PBS containing 2% donkey serum and 1% BSA at room temperature for 2 h. The primary antibodies: anti‐LYVE‐1 (ab14917, Abcam, 1:200) were used to perform incubation overnight at 4 °C. Meninges were washed in PBS‐Tween‐20 (PBS‐T) for 10 min and 3 times and then were submerged in secondary antibody donkey anti‐rabbit Alexa Flour 488 (ab150073, Abcam, 1:500) for 2 h in room temperature. After PBS‐T washing and DAPI labeling, meninges were mounted with a mounting medium.

### Micro‐Computed Tomography Examination

The structural changes of the skull defect were analyzed using micro‐CT (Scanco Medical, Switzerland). Briefly, the skull was fixed in a custom‐made holder. Image acquisition was performed at 70 kV and 118 µA, with a resolution of 18 µm per voxel. The gray‐scale images were segmented to perform 3D reconstruction of the mineralized bone phase to quantify the microarchitecture of trabecular and cortical bone using the software of the micro‐CT workstation. For skull defect analysis, 50 continuous slices were selected as the volume of interest (VOI). Mineralized tissues were constructed using thresholds between 211 and 1000. BV/TV and BMD were analyzed using the build‐in program (Image Processing Language v4.29d, Scanco Medical).

### Hematoxylin and Eosin Staining (H &E Staining)

All skulls were fixed in 10% formalin for 24 hours at room temperature and then subject to decalcification in 10% ethylenediaminetetraacetic acid (EDTA) solution (pH 7.2) for four weeks with intermittent shaking before embedding in paraffin; 5 µm sections were cut by a rotary microtome (HM 355 S, ThermoFisher Scientific, Germany) along the coronal plane and perpendicular to sagittal sutures. Sections were deparaffinized in xylene (mixed isomers) 2 times for 5 min each; then rehydrated through 70% alcohol 10 s, 80% alcohol 10 s, 90% alcohol 10 s for 2 times, 100% alcohol 3min+5min, then cleared in 3 changes of Xylene 1 min each change and mounted with DPX coverslip.

### Masson Staining

All skulls were fixed in 10% formalin for 24 h at room temperature and then subject to decalcification in 10% ethylenediaminetetraacetic acid (EDTA) solution (pH 7.2) for four weeks with intermittent shaking before embedding in paraffin; 5 µm sections were cut by a rotary microtome (HM 355 S, ThermoFisher Scientific, Germany) along the coronal plane and perpendicular to sagittal sutures. Sections were deparaffinized in xylene (mixed isomers) 2 times for 5 min each, then stained with Trichrome Stain Kit (Connective Tissue Stain) (Abcam, ab150686) according to the manufacturer's instructions.

### Immunohistochemistry Staining

All skulls were fixed in 10% formalin for 24 h at room temperature and then subject to decalcification in 10% ethylenediaminetetraacetic acid (EDTA) solution (pH 7.2) for four weeks with intermittent shaking before embedding in paraffin; 5 µm sections were cut by a rotary microtome (HM 355 S, ThermoFisher Scientific, Germany) along the coronal plane and perpendicular to sagittal sutures. Sections were deparaffinized in xylene (mixed isomers) 2 times for 5 min each; then rehydrated in 100% alcohol for 3 min, 90% alcohol for 2 min, and 70% alcohol for 2 min, and in deionized H_2_O. The endogenous peroxidase activity is demolished with 3% H2O2 in water or methanol for 20 min. To reduce non‐specific hydrophobic interactions between the primary antibodies and the tissue, the sections are treated with serum blocking reagent for 30 min. Then, the primary antibodies of anti‐OCN (MAB1419, R&D Systems, 1:200) and anti‐COL‐1 (Ab270993, Abcam, 1:200) are incubated with the section at 4°C overnight. After washing with the buffered PBS secondary antibodies anti‐mouse Peroxidase Conjugated (Rockland, 610–1319,1:500) and anti‐goat Peroxidase Conjugated (Rockland, 611–1302,1:500) are applied for 60 min. After rinsing the slides with buffered PBS, the DAB/AEC chromogen solution is applied to cover the entire section and incubated for 3–20 min. The intensity of color development is monitored under a light microscope. The slides are then washed with buffer 3 times for 10 min each. The sections are finally stained with hematoxylin, dehydrated through graded alcohol (70% alcohol 10 s, 80% alcohol 10 s, 90% alcohol 10 s for 2 times, 100% alcohol 3min+5min, xylene 3min+5min) and mounted with DPX coverslip.

### Image Analysis

Images were acquired using a confocal microscope (Carl Zeiss LSM 880 Laser Confocal). Quantitative measurements were performed using ImageJ software (NIH, Bethesda, Maryland, USA). For quantification of neurons in Nissl staining, all images were evaluated by a co‐author who was blinded to the group assignment. Three inconsecutive sections per brain were randomly chosen and 5 samples in each group were evaluated, to calculate the average number of neurons in each group. For evaluation of the area of brain infarction, four sections (bregma 1.50 mm, ‐0.25 mm, ‐2.00 mm, and ‐3.75 mm, respectively) from each brain were collected for evaluation of the area of brain infarction. The infarct area fraction (%) was calculated as the proportion of the ipsilateral infarct area to the contralateral hemisphere area. Quantification of injected OVA‐A647 was defined as the ratio of the area of OVA‐A647 to the whole area of dCLNs, brain, or lymphatic vessels. Quantification of expression LYVE‐1, NeuN, GFAP, Iba1, P‐tau, and CD31 was determined by the number of positive vessels or cells or positive areas in each field. For each sample, at least 3 histological sections were quantified. To quantify the diameter of lymphatic vessels, 5 random locations were chosen from each image, diameters were measured and then the mean value was calculated. For Sholl analysis, the number of Iba1 + branches intersecting with a radius of 0–60 µm from the soma was calculated by the average of 36 microglia per group (3 Iba1^+^ cells per section, 3 sections per rat, 6 rats per group).

### Nissl Staining

Cresyl violet stain solution was prepared with 0.1 g cresyl violet acetate (CX2065, Sigma‐Aldrich, USA) in 100 mL ddH2O and 250 µL glacial acetic acid. Frozen brain sections were rinsed in PBS and stained with cresyl violet solution for 20 mins. After dehydration, sections were mounted with DPX mountant (44581, Sigma‐Aldrich, USA), and the histological images were acquired using the microscope (DM5500, Leica Microsystems, Germany).

### Behavioral Tests

*Y Maze*: Y maze is a behavior test that assesses spatial learning and memory ability in rodents. Y maze consists of three arms (length 50 cm, width 16 cm, height 32 cm), which are positioned at 120 degrees to each other and form a “Y” shape. The protocol of the Y maze was conducted according to a previous publication. Different pictures of colors or shapes were used to help the rat recognize each arm. The testing environment in the laboratory is kept quiet and direct sunlight should be avoided. The rats were brought from the animal room into the behavioral laboratory 1 h before the test. All arms of the Y‐maze were cleaned before proceeding to the next rat.

*Spontaneous or Continuous Alternation Test*: Spontaneous alteration is used as a measurement of working memory, which is a test to study the willingness of rats to explore new environments. The hippocampus, septum, basal forebrain, and prefrontal cortex are associated with this ability. The arms of the maze are labeled as A, B, and C. The Rat was placed on the tail of one of the arms of the Y maze and allowed to explore freely for 8 min, which was recorded by a camera. The following parameters were calculated and measured: (1) the total number of entries: The number of times the animals entered the arms of the maze (An entry is defined as the rat entering an arm with four paws). (2) an alternation: successively enter all three arms of the Y‐maze consecutively, for instance, an A‐C‐B‐C‐A consists of two alternations. Therefore, spontaneous alternation behavior score = [total number of alternations / (total number of entries – 2)] * 100%.

*Spatial Reference Memory*: The rat was first trained as follows: one of the arms was closed with a baffle, the others were opened; the rats were put into the other two arms by placing the rats facing the center and recording which arm the animals have been placed in, then let them move freely for 10 min. For the testing, 1 h after the training, the baffle was removed, and the rats were placed in the same arm that was used in the previous run, and let the rats explore freely for 5 min, then the movement distance and time spent in each arm were recorded.

*Novel Object Recognition Test*: NOR is a behavior test applied to evaluate cognition, particularly recognition memory in rodents. The test is based on the rodents' natural proclivity for exploring novelty. The protocol was designed as previously published. Rats were presented with two same objects in the first session and one of them was replaced by a new one in the second session. To reduce novelty‐induced stress, we handled each of the rats twice a week and 1 min for once. All rats were placed into the experiment room 30 min before performing the test. In the first part, rats were put into a square box (length 43 cm, width 43 cm, height 30 cm) with two same objects (two green cuboid objects) for 10 mins and were recorded by a camera. The cuboid objects were put in the opposite corners of a square. Paper tissue and alcohol were used to clean the box between each test. After 1 h, one of the objects in the box was replaced by a red one and the trail of rats in the box for 10 mins again. Then the amount of time taken to explore the old object and the new object was calculated.

*Catwalk Gait Analysis*: A glass walkway (109 × 15 × 0.6 cm, L × W × H) is illuminated with a green light below and the distance between the beams. The light reflected the paws when rats walked across the glass walkway and a high‐speed video camera recorded the completed runs. An intensity threshold was set to 0.16, the camera gain was set to 28, and the maximum allowed speed variation was set to 60%. All the experiments were performed in the same environment and by the same investigator. Three complete walks that were finished in the same direction (from left to right of the glass walkway) were recorded. The following parameters of the right front paw (RF), right hind paw (RH), left front paw (LF), and left hind paw (LH) of all rats were recorded by the computer, including print area, intensity, stride length, and walking speed. Rats were subjected to gait analysis at days 3 (baseline), 8, and 13 after CBT surgery.

*Rotarod Test*: Rotarod tests were performed to evaluate the motor function of rats by using an accelerating rotarod (ITC Life Science Inc. CA, USA). Rats were trained for 3 days with 3 trials per day before doing the tests to obtain stable baselines. The initial speed of the rod was 10 rounds per min, and it gradually speeded up to 30 rounds min^−1^ after 100 s. Only rats that stayed on the rods for 80–100 s were included for further experiments. A trial ended if the animal fell off from the rods or gripped the device and spun around for two consecutive rotations without attempting to walk on the rod. Durations of three trails were collected for data analysis. Rats were subjected to rotarod tests on days 3 (baseline), 8, and 13 after CBT surgery.

### Statistical Analysis

Statistical analysis was performed using GraphPad Prism (Version 8.0.1, CA, USA). Data are presented as mean ± standard deviation (SD). The normal distribution of data was first checked with the Anderson‐Darling, D'Agostino, and Shapiro‐Wilk normality tests. One‐way or two‐way analysis of variance (ANOVA) with Bonferroni's post hoc tests were used for multiple comparisons among three or more groups. P value <0.05 was considered statistically significant.

## Conflict of Interest

The authors declare no conflict of interest.

## Supporting information



Supporting Information

## Data Availability

All requests for raw and analyzed data and materials are promptly reviewed by the Technology Licensing Office of the Chinese University of Hong Kong to verify whether the request is subject to any intellectual property or confidentiality obligations. Any data and materials that can be shared will be released via a Material Transfer Agreement.

## References

[1] K. Dams‐O'Connor , S. B. Juengst , J. Bogner , N. D. Chiaravalloti , J. D. Corrigan , J. T. Giacino , C. L. Harrison‐Felix , J. M. Hoffman , J. M. Ketchum , A. H. Lequerica , J. H. Marwitz , A. C. Miller , R. Nakase‐Richardson , A. R. Rabinowitz , A. M. Sander , R. Zafonte , F. M. Hammond , Lancet Neurol. 2023, 22, 517. 37086742 10.1016/S1474-4422(23)00065-0

[2] A. I. R. Maas , D. K. Menon , G. T. Manley , M. Abrams , C. Akerlund , N. Andelic , M. Aries , T. Bashford , M. J. Bell , Y. G. Bodien , B. L. Brett , A. Buki , R. M. Chesnut , G. Citerio , D. Clark , B. Clasby , D. J. Cooper , E. Czeiter , M. Czosnyka , K. Dams‐O'Connor , V. De Keyser , R. Diaz‐Arrastia , A. Ercole , T. A. van Essen , E. Falvey , A. R. Ferguson , A. Figaji , M. Fitzgerald , B. Foreman , D. Gantner , et al., Lancet Neurol. 2022, 21, 1004.36183712

[3] T. V. Veenith , E. L. Carter , T. Geeraerts , J. Grossac , V. F. Newcombe , J. Outtrim , G. S. Gee , V. Lupson , R. Smith , F. I. Aigbirhio , T. D. Fryer , Y. T. Hong , D. K. Menon , J. P. Coles , JAMA Neurol. 2016, 73, 542. 27019039 10.1001/jamaneurol.2016.0091

[4] T. M. Andriessen , B. Jacobs , P. E. Vos , J. Cell. Mol. Med. 2010, 14, 2381. 20738443 10.1111/j.1582-4934.2010.01164.x PMC3823156

[5] B. L. Brett , R. C. Gardner , J. Godbout , K. Dams‐O'Connor , C. D. Keene , Biol. Psychiatry 2022, 91, 498. 34364650 10.1016/j.biopsych.2021.05.025 PMC8636548

[6] P. K. Crane , L. E. Gibbons , K. Dams‐O'Connor , E. Trittschuh , J. B. Leverenz , C. D. Keene , J. Sonnen , T. J. Montine , D. A. Bennett , S. Leurgans , J. A. Schneider , E. B. Larson , JAMA Neurol. 2016, 73, 1062. 27400367 10.1001/jamaneurol.2016.1948 PMC5319642

[7] A. C. Bolte , A. B. Dutta , M. E. Hurt , I. Smirnov , M. A. Kovacs , C. A. McKee , H. E. Ennerfelt , D. Shapiro , B. H. Nguyen , E. L. Frost , C. R. Lammert , J. Kipnis , J. R. Lukens , Nat. Commun. 2020, 11, 4524. 32913280 10.1038/s41467-020-18113-4 PMC7483525

[8] J. J. Iliff , M. J. Chen , B. A. Plog , D. M. Zeppenfeld , M. Soltero , L. Yang , I. Singh , R. Deane , M. Nedergaard , J. Neurosci. 2014, 34, 16180. 25471560 10.1523/JNEUROSCI.3020-14.2014 PMC4252540

[9] D. J. Cooper , J. V. Rosenfeld , L. Murray , Y. M. Arabi , A. R. Davies , P. D'Urso , T. Kossmann , J. Ponsford , I. Seppelt , P. Reilly , R. Wolfe , D. T. Investigators , N. Engl. J. Med. 2011, 364, 1493. 21434843 10.1056/NEJMoa1102077

[10] D. W. Marion , Lancet Neurol. 2011, 10, 497. 21601156 10.1016/S1474-4422(11)70098-9

[11] B. Ozoner , Curr. Neurol. Neurosci. Rep. 2021, 21, 62. 34674047 10.1007/s11910-021-01147-6

[12] J. C. Eaton , M. E. Greil , D. Nistal , D. J. Caldwell , E. Robinson , Z. Aljuboori , N. Temkin , R. H. Bonow , R. M. Chesnut , J. Neurosurg. 2022, 137, 776. 35061995 10.3171/2021.11.JNS211557

[13] B. Tinterri , G. Capo , S. Chibbaro , M. Ganau , D. Cannizzaro , I. Zaed , Neurosurgery 2022, 90, 50. 10.1227/NEU.0000000000001792 34995273

[14] F. P. Koch , M. M. Yuhasz , R. Travieso , K. Wong , J. Clune , Z. W. Zuang , D. M. Steinbacher , Plast. Reconstr. Surg. 2013, 131, 453e. 23446610 10.1097/PRS.0b013e31827c724e

[15] F. P. Koch , M. M. Yuhasz , R. Travieso , K. Wong , J. Clune , Z. W. Zhuang , J. Van Houten , D. M. Steinbacher , J. Craniofacial Surg. 2014, 25, 766. 10.1097/SCS.0000000000000769 24820707

[16] M. E. Elsalanty , I. Zakhary , S. Akeel , B. Benson , T. Mulone , G. R. Triplett , L. A. Opperman , Ann. Plast. Surg. 2009, 63, 441. 19770704 10.1097/SAP.0b013e31818d130c PMC2811127

[17] M. E. Elsalanty , T. N. Taher , I. E. Zakhary , O. A. Al‐Shahaat , M. Refai , H. A. El‐Mekkawi , J. Craniofacial Surg. 2007, 18, 1397. 10.1097/scs.0b013e31814fb593 17993888

[18] L.‐c. Kong , H. A. Li , Q.‐l. Kang , G. Li , J. Orthop. Res. 2020, 25, 3.

[19] G. Li , Current Opinion in Orthopaedics 2004, 15, 325.

[20] S. Bai , X. Lu , Q. Pan , B. Wang , K. Pong U , Y. Yang , H. Wang , S. Lin , L. Feng , Y. Wang , Y. Li , W. Lin , Y. Wang , X. Zhang , Y. Li , L. Li , Z. Yang , M. Wang , W. Y. Lee , X. Jiang , G. Li , Stroke 2022, 53, 1373. 35135326 10.1161/STROKEAHA.121.037912

[21] H. M. Bramlett , W. D. Dietrich , J Cereb Blood Flow Metab 2004, 24, 133. 14747740 10.1097/01.WCB.0000111614.19196.04

[22] A. Kunz , U. Dirnagl , P. Mergenthaler , Best Pract. Res. Clin. Anaesthesiol. 2010, 24, 495. 21619862 10.1016/j.bpa.2010.10.001

[23] G. Edwards III, J. Zhao , P. K. Dash , C. Soto , I. Moreno‐Gonzalez , J. Neurotrauma. 2020, 37, 80. 31317824 10.1089/neu.2018.6348 PMC6921297

[24] R. Rubenstein , B. Chang , J. K. Yue , A. Chiu , E. A. Winkler , A. M. Puccio , R. Diaz‐Arrastia , E. L. Yuh , P. Mukherjee , A. B. Valadka , JAMA neurology 2017, 74, 1063. 28738126 10.1001/jamaneurol.2017.0655 PMC5710183

[25] O. Albayram , A. Kondo , R. Mannix , C. Smith , C.‐Y. Tsai , C. Li , M. K. Herbert , J. Qiu , M. Monuteaux , J. Driver , Nat. Commun. 2017, 8, 1000. 29042562 10.1038/s41467-017-01068-4 PMC5645414

[26] W. J. Yang , W. Chen , L. Chen , Y. J. Guo , J. S. Zeng , G. Y. Li , W. S. Tong , Acta Neurol. Scand. 2017, 135, 622. 27439764 10.1111/ane.12644

[27] J. E. Burda , A. M. Bernstein , M. V. Sofroniew , Exp. Neurol. 2016, 275, 305. 25828533 10.1016/j.expneurol.2015.03.020 PMC4586307

[28] A. F. Ramlackhansingh , D. J. Brooks , R. J. Greenwood , S. K. Bose , F. E. Turkheimer , K. M. Kinnunen , S. Gentleman , R. A. Heckemann , K. Gunanayagam , G. Gelosa , Ann. Neurol. 2011, 70, 374. 21710619 10.1002/ana.22455

[29] J. W. Breslin , N. Gaudreault , K. D. Watson , R. Reynoso , S. Y. Yuan , M. H. Wu , Am. J. Physiol. Heart Circ. Physiol. 2007, 293, H709. 17400713 10.1152/ajpheart.00102.2007

[30] J. Chen , L. Wang , H. Xu , L. Xing , Z. Zhuang , Y. Zheng , X. Li , C. Wang , S. Chen , Z. Guo , Nat. Commun. 2020, 11, 3159. 32572022 10.1038/s41467-020-16851-z PMC7308412

[31] A. Apaydin , B. Yazdirduyev , T. Can , N. Keklikoglu , Int. J. Oral Maxillofac. Surg. 2011, 40, 408. 21195586 10.1016/j.ijom.2010.11.007

[32] R. J. Mackool , R. A. Hopper , B. H. Grayson , R. Holliday , J. G. McCarthy , Plast. Reconst. Surg. 2003, 111, 1804. 12711939 10.1097/01.PRS.0000055431.19215.0A

[33] H.‐Q. Wang , X.‐K. Li , Z.‐X. Wu , Y.‐Y. Wei , Z.‐J. Luo , BMC Musculoskelet. Disord. 2008, 9, 101. 18611283 10.1186/1471-2474-9-101 PMC2483275

[34] N. M. Rowe , B. J. Mehrara , J. S. Luchs , M. E. Dudziak , D. S. Steinbrech , P. B. Illei , G. J. Fernandez , G. K. Gittes , M. T. Longaker , Ann. Plast. Surg. 1999, 42, 470. 10340853 10.1097/00000637-199905000-00002

[35] Y. Yang , Y. Li , Q. Pan , S. Bai , H. Wang , X. H. Pan , K. K. Ling , G. Li , Bone Joint Res. 2022, 11, 189. 35358393 10.1302/2046-3758.114.BJR-2021-0364.R1 PMC9057526

[36] X. Hu , Q. Deng , L. Ma , Q. Li , Y. Chen , Y. Liao , F. Zhou , C. Zhang , L. Shao , J. Feng , T. He , W. Ning , Y. Kong , Y. Huo , A. He , B. Liu , J. Zhang , R. Adams , Y. He , F. Tang , X. Bian , J. Luo , Cell Res. 2020, 30, 229. 32094452 10.1038/s41422-020-0287-8 PMC7054407

[37] J. H. Ahn , H. Cho , J.‐H. Kim , S. H. Kim , J.‐S. Ham , I. Park , S. H. Suh , S. P. Hong , J.‐H. Song , Y.‐K. Hong , Nature 2019, 572, 62. 31341278 10.1038/s41586-019-1419-5

[38] X. Li , L. Qi , D. Yang , S. Hao , F. Zhang , X. Zhu , Y. Sun , C. Chen , J. Ye , J. Yang , Nat. Neurosci. 2022, 25, 577. 35524140 10.1038/s41593-022-01063-z

[39] A. Louveau , I. Smirnov , T. J. Keyes , J. D. Eccles , S. J. Rouhani , J. D. Peske , N. C. Derecki , D. Castle , J. W. Mandell , K. S. Lee , Nature 2015, 523, 337. 26030524 10.1038/nature14432 PMC4506234

[40] J. Liao , M. Zhang , Z. Shi , H. Lu , L. Wang , W. Fan , X. Tong , H. Yan , J. Neurotrauma. 2023, 40, 383. 36106596 10.1089/neu.2022.0150

[41] F. Gonzalez‐Ortiz , M. Dulewicz , N. J. Ashton , P. R. Kac , H. Zetterberg , E. Andersson , Y. Yakoub , J. Hanrieder , M. Turton , P. Harrison , JAMA Network Open 2023, 6, 2321554. 10.1001/jamanetworkopen.2023.21554 PMC10318474 37399012

[42] W. Zou , T. Pu , W. Feng , M. Lu , Y. Zheng , R. Du , M. Xiao , G. Hu , Transl. Neurodegener 2019, 8, 7. 30867902 10.1186/s40035-019-0147-y PMC6396507

[43] S.‐J. Hsu , C. Zhang , J. Jeong , S.‐i. Lee , M. McConnell , T. Utsumi , Y. Iwakiri , Gastroenterology 2021, 160, 1315. 33227282 10.1053/j.gastro.2020.11.036 PMC7956141

[44] O. N. Kokiko‐Cochran , J.P. Godbout. Front. immunol. 2018, 9, 672. 10.3389/fimmu.2018.00672 PMC5900037 29686672

[45] M. Jin , H. Shiwaku , H. Tanaka , T. Obita , S. Ohuchi , Y. Yoshioka , X. jin , K. Kondo , K. Fujita , H. Homma , K. Nakajima , M. Mizuguchi , H. Okazawa , Nat. Commun. 2021, 12, 6565. 34782623 10.1038/s41467-021-26851-2 PMC8592984

[46] A. Olate‐Briones , E. Escalona , C. Salazar , M. J. Herrada , C. Liu , A. A. Herrada , N. Escobedo , FASEB J. 2022, 36, 22276. 10.1096/fj.202101574RR 35344212

[47] S. D. Mesquita , Z. Papadopoulos , T. Dykstra , L. Brase , F. G. Farias , M. Wall , H. Jiang , C. D. Kodira , K. A. Lima , J. Herz , A. Louveau , D. H. Goldman , A. F. Salvador , S. Onengut‐Gumuscu , E. Farber , N. Dabhi , T. Kennedy , M. G. Milam , W. Baker , I. Smirnov , S. S. Rich , D. I. A. Network , B. A. Benitez , C. M. Karch , R. J. Perrin , M. Farlow , J. P. Chhatwal , D. M. Holtzman , C. Cruchaga , O. Harari , et al., Nature 2021, 593, 255. 33911285 10.1038/s41586-021-03489-0 PMC8817786

[48] E. Song , T. Mao , H. Dong , L. S. B. Boisserand , S. Antila , M. Bosenberg , K. Alitalo , J.‐L. Thomas , A. Iwasaki , Nature 2020, 577, 689. 31942068 10.1038/s41586-019-1912-x PMC7100608

[49] K. A. Jacobsen , Z. S. Al‐Aql , C. Wan , J. L. Fitch , S. N. Stapleton , Z. D. Mason , R. M. Cole , S. R. Gilbert , T. L. Clemens , E. F. Morgan , J. Bone Miner. Res. 2008, 23, 596. 18433297 10.1359/JBMR.080103 PMC2674537

[50] Z. Ai‐Aql , A. S. Alagl , D. T. Graves , L. C. Gerstenfeld , T. A. Einhorn , J. Dent. Res. 2008, 87, 107. 18218835 10.1177/154405910808700215 PMC3109437

[51] S. A. Stacker , M. G. Achen , Biomolecules 2018, 8, 1. 29300337 10.3390/biom8010001 PMC5871970

[52] S. Yang , N. Wang , Y. Ma , S. Guo , S. Guo , H. Sun , Int. J. Oral Sci. 2022, 14, 4. 35067679 10.1038/s41368-021-00156-y PMC8784536

[53] A. G. Kolias , P. J. Kirkpatrick , P. J. Hutchinson , Nat. Rev. Neurol. 2013, 9, 405. 23752906 10.1038/nrneurol.2013.106

[54] C. Szpalski , J. Barr , M. Wetterau , P. B. Saadeh , S. M. Warren , Neurosurg. Focus. 2010, 29, E8. 10.3171/2010.9.FOCUS10201 21121722

[55] Z. Li , J. Liu , C. Li , M. Wu , Y. Li , Y. Cui , W. Xiong , F. Yang , B. Liu , Orthop. Surg. 2023, 15, 3046. 37963829 10.1111/os.13936 PMC10694017

[56] P. A. Gerety , J. D. Wink , R. D. Sherif , N. Clarke , H. D. Nah , J. A. Taylor , J. Craniofacial Surg. 2014, 25, 1917. 10.1097/SCS.0000000000000987 25119411

[57] L. G. González‐Bonet , World Neurosurg 2021, 147, 67. 33359522 10.1016/j.wneu.2020.12.056

[58] C. A. Almendárez‐Sánchez , E. Reyna‐Martínez , A. Vara‐Castillo , M. I. Ruiz‐Flores , L. Álvarez‐Vázquez , S. Solorio‐Pineda , A. A. Tafur‐Grandett , A. Sosa‐Nájera , J. A. Franco‐Jiménez , Interdiscip. Neurosurg. 2021, 26, 101311.

[59] N. C. Cabbad , M. W. Stalder , A. Arroyave , E. M. Wolfe , S. A. Wolfe , Plast. Reconst. Surg. 2019, 143, 1713. 31136489 10.1097/PRS.0000000000005677

[60] C. L. Rosinski , A. N. Chaker , J. Zakrzewski , B. Geever , S. Patel , R. G. Chiu , D. M. Rosenberg , R. Parola , K. Shah , M. Behbahani , World Neurosurg 2019, 131, 312. 10.1016/j.wneu.2019.07.139 31351936

[61] J. A. Goldstein , J. T. Paliga , S. P. Bartlett , Curr. Opin. Otolaryngol Head Neck. Surg. 2013, 21, 400. 23770828 10.1097/MOO.0b013e328363003e

[62] J. Mora‐Macías , A. Pajares , P. Miranda , J. Domínguez , E. Reina‐Romo , J. Mech. Behav. Biomed. Mater. 2017, 74, 236. 28623826 10.1016/j.jmbbm.2017.05.031

[63] M. P. Yavropoulou , J. Yovos , J. Musculoskelet Neuronal Interact. 2016, 16, 221. 27609037 PMC5114345

[64] W. R. Thompson , C. T. Rubin , J. Rubin , Gene 2012, 503, 179. 22575727 10.1016/j.gene.2012.04.076 PMC3371109

[65] S. M. Mantila Roosa , Y. Liu , C. H. Turner , J. Bone Miner. Res. 2011, 26, 100. 20658561 10.1002/jbmr.193 PMC3179310

[66] C. da Silva Madaleno , J. Jatzlau , P. Knaus , Bone 2020, 137, 115416. 32422297 10.1016/j.bone.2020.115416

[67] Z. Jin , Z. Chen , T. Liang , W. Liu , Z. Shan , D. Tan , J. Chen , J. Hu , L. Qin , J. Xu , J. Orthop. Translat. 2025, 50, 71. 39868349 10.1016/j.jot.2024.10.008 PMC11763218

[68] M. F. Paccione , B. J. Mehrara , S. M. Warren , J. A. Greenwald , J. A. Spector , J. S. Luchs , M. T. Longaker , J. Craniofacial Surg. 2001, 12, 175. 10.1097/00001665-200103000-00015 11314629

[69] R. Tazawa , H. Minehara , T. Matsuura , T. Kawamura , K. Uchida , G. Inoue , W. Saito , M. Takaso , Biomed Res. Int. 2019, 2019, 1. 10.1155/2019/1014594 PMC6948306 31950029

[70] V. Kirkin , W. Thiele , P. Baumann , R. Mazitschek , K. Rohde , G. Fellbrich , H. Weich , J. Waltenberger , A. Giannis , J. P. Sleeman , Int. J. Cancer 2004, 112, 986. 15386354 10.1002/ijc.20509

[71] R. Benedito , S. F. Rocha , M. Woeste , M. Zamykal , F. Radtke , O. Casanovas , A. Duarte , B. Pytowski , R. H. Adams , Nature 2012, 484, 110. 22426001 10.1038/nature10908

[72] J. Wang , J. Yu , H. Wei , A. Wang , Z. Hou , M. Kumi , X. Wang , T. Wang , P. Li , W. Huang , Adv. Funct. Mater. 2024, 34, 2407394.

[73] N. D. Osier , C. E. Dixon , Front. Neurol. 2016, 7, 134. 27582726 10.3389/fneur.2016.00134 PMC4987613

