# Supplementary material for: A Novel Cranial Bone Transport Technique Repairs Skull Defect and Minimizes Brain Injury Outcome in Traumatic Brain Injury Rats
Source: Adv Sci (Weinh). 2025 May 31;12(32):e04467. doi: 10.1002/advs.202504467 (PMC12407380; doi:10.1002/advs.202504467)
Supplement: Supplementary file 1 — Supporting Information [file ADVS-12-e04467-s001.docx]

**Supplemental Information**

**A novel cranial bone transport technique repairs skull defect and minimizes brain injury outcome in traumatic brain injury rats**


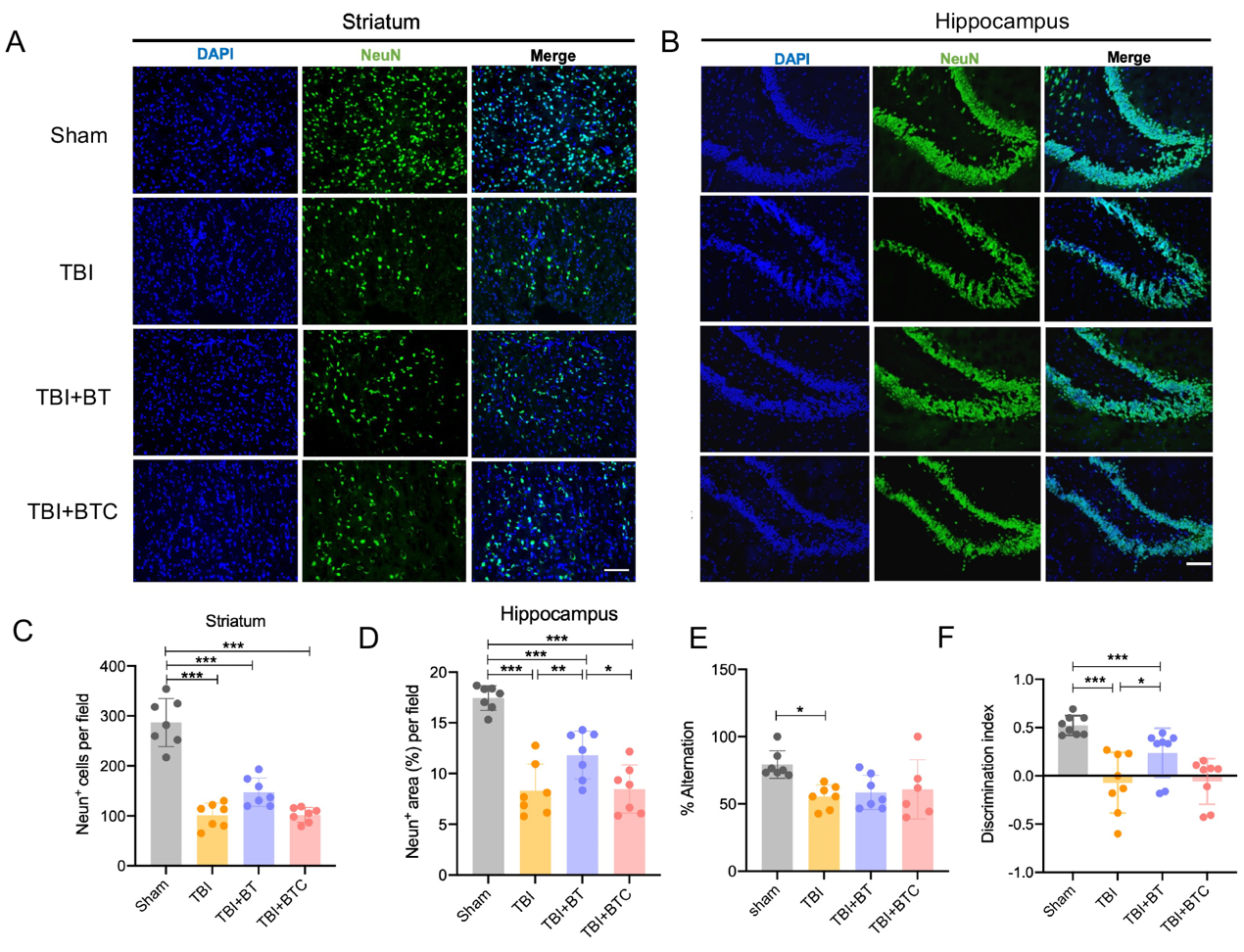


**Supplemental Figure 1 CBT attenuated neuronal death (striatum, hippocampus) and improved cognitive function in TBI rats.** (**A**) Representative image of perilesional striatum section showing NeuN+ immunofluorescence at 14 days after TBI. (**B**) Representative image of perilesional hippocampus section showing NeuN+ immunofluorescence at 14 days after TBI. (**C**) Quantification of total viable neurons in the perilesional striatum. (**D**) Quantification of area (%) of NeuN+ positive cells in the hippocampus. (**E**) Histograms represent the alternation percentage for each experimental group. (**F**) Histograms represent the discrimination index for each experimental group. Discrimination index = (time spent with novel object − time spent with old object)/total exploration time. *p<0.05, **p<0.01, ***p<0.001. Data are shown as mean ± SD.


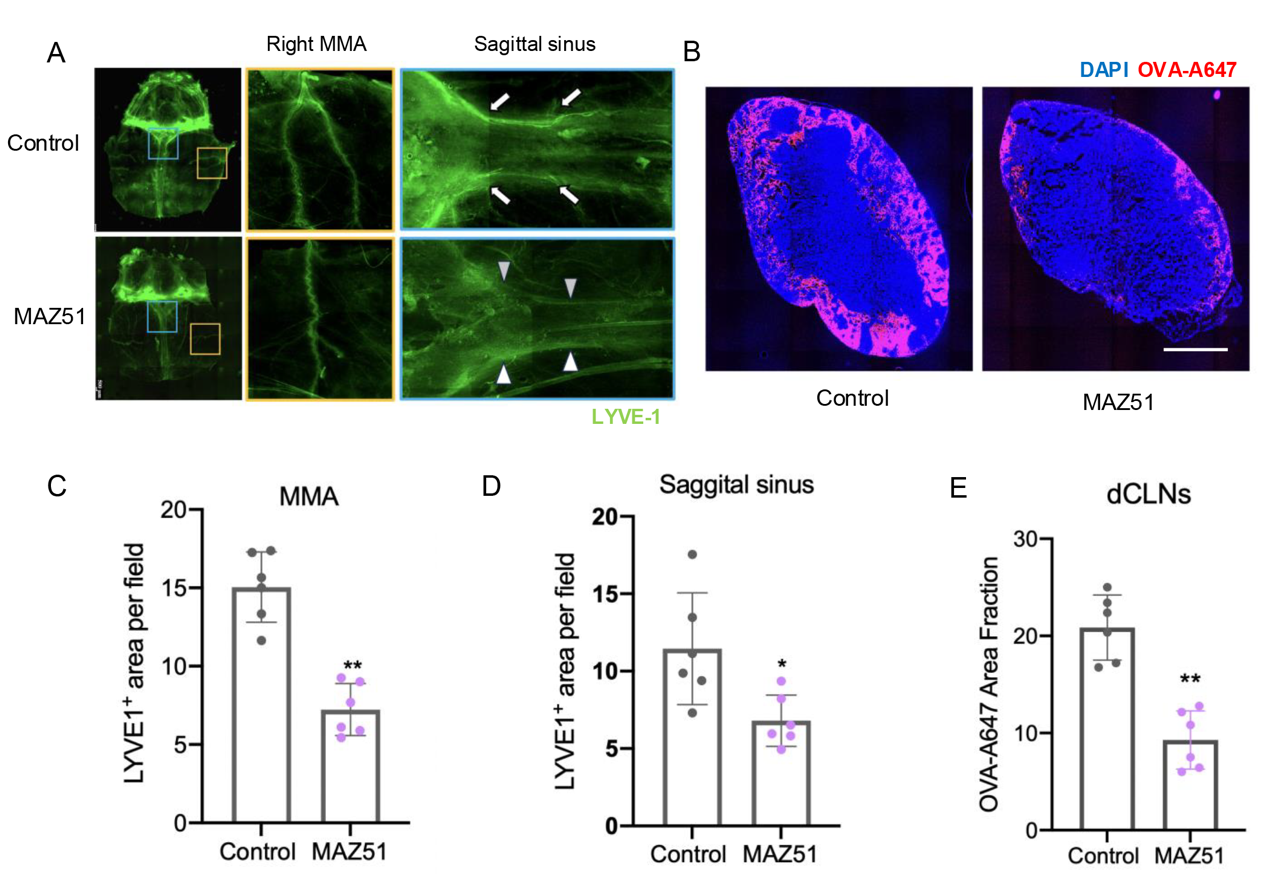


**Supplemental Figure 2. MAZ51 reduced MLVs coverage and ablated meningeal lymphatic draining function in rats.** (**A**) Representative images of MLVs in MMA region (yellow box) and sagittal sinus region (blue box) stained with LYVE-1 (green) in control group (white arrows) and MAZ51 group (white triangles). (**B**) Representative images of dCLNs with OVA-A647 (red) stained with DAPI (blue), scale bar=500μm. (**C**) Quantification of MLVs coverage in MMA(middle cerebral artery) region. (**D**) Quantification of MLVs coverage in sagittal sinus region. (**E**) Quantification of OVA-A647 in dCLNs in two groups. **p*<0.05, ***p*<0.01, ****p*<0.001. Data are shown as mean ± SD.


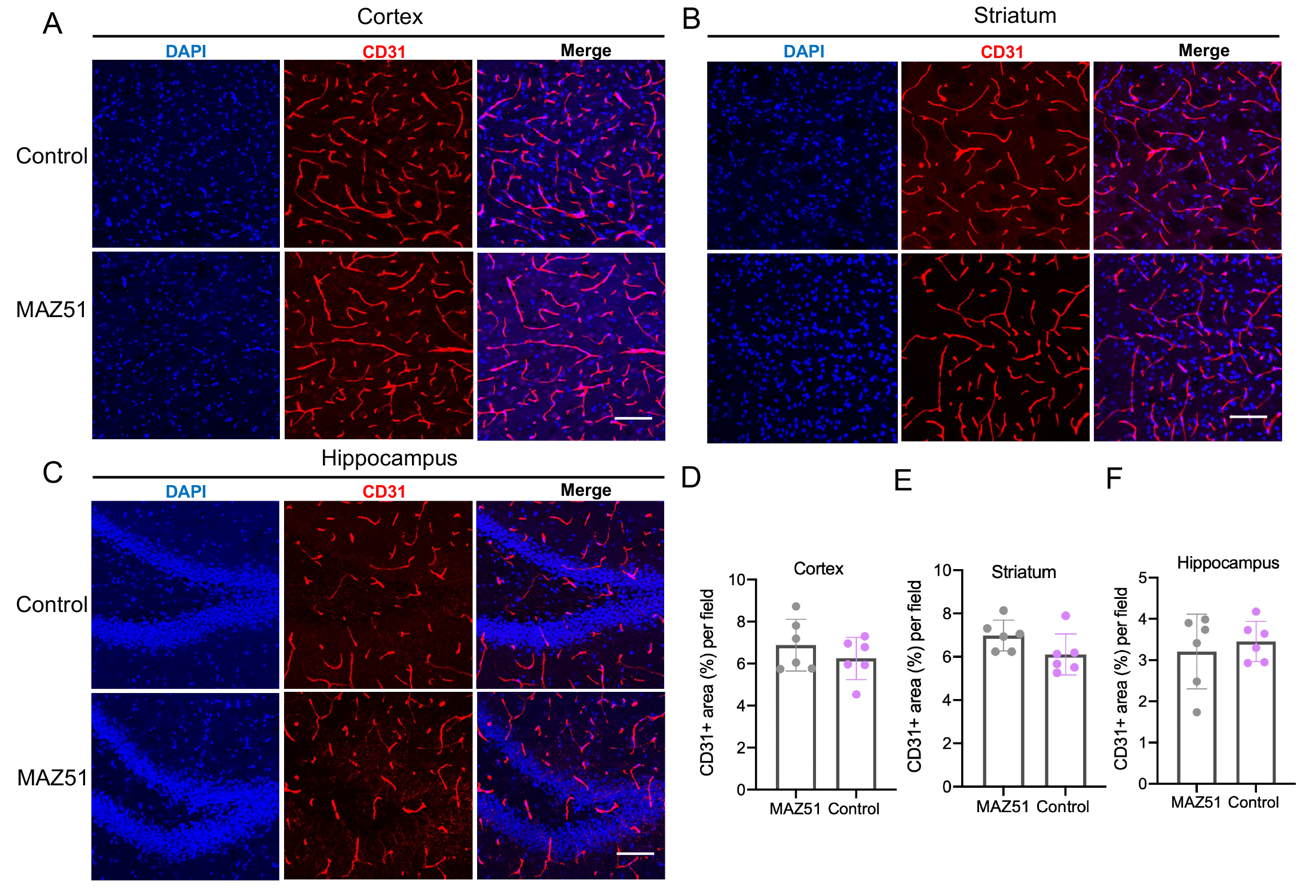


**Supplemental Figure 3 The ablation of meningeal lymphatics by MAZ51 did not alter the cerebral blood vessels. (A**) Representative image of perilesional cortex section showing CD31+ immunofluorescence after MAZ51 injection. (**B**) Representative image of perilesional striatum section showing CD31+ immunofluorescence after MAZ51 injection. (**C**) Representative image of perilesional hippocampus section showing CD31+ immunofluorescence after MAZ51 injection. (**D**) Quantification of CD31+ positive area (%) in the cortex. (**E**) Quantification of CD31+ positive area (%) in the striatum. (**F**) Quantification of CD31+ positive area (%) in the hippocampus. *p<0.05, **p<0.01, ***p<0.001. Data are shown as mean ± SD.


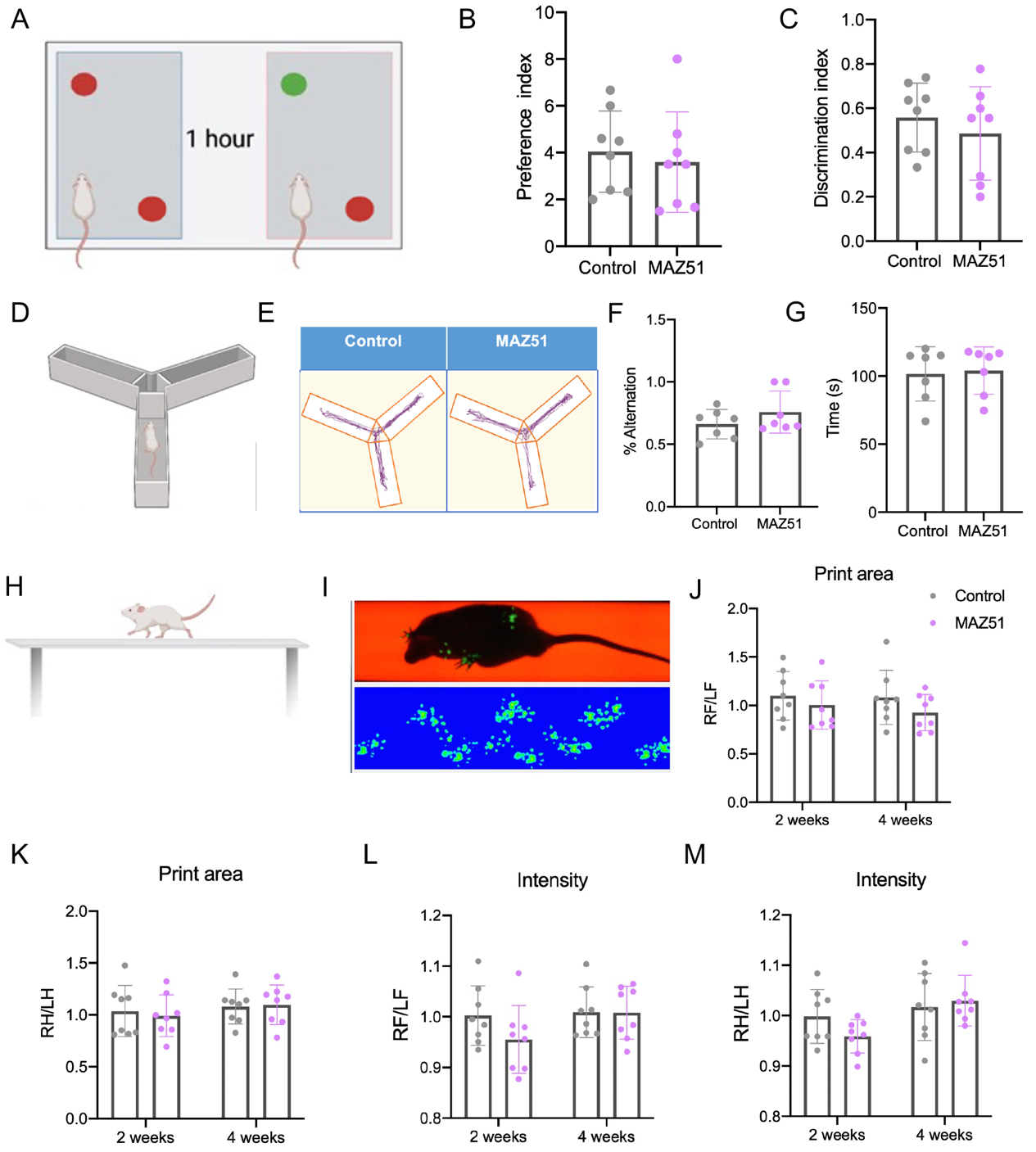


**Supplemental Figure 4 The ablation of meningeal lymphatics by MAZ51 did not alter the motor, memory and cognitive function of rats. (A**) Schematic of the novel objection test. (**B**) Histograms represent the preference index for each experimental group. Preference index=time spent with novel object/total exploration time. (**C**) Histograms represent the discrimination index for each experimental group. Discrimination index = (time spent with novel object − time spent with old object)/total exploration time. (**D**) Schematic of the Y maze test. (**E**) Representative images of track in spatial memory test part for two groups. (**F**) Histograms represent the alternation percentage for each experimental group. (**G**) Histograms represent the duration in B arm for each experimental group. **(H**) Schematic of the catwalk gait analysis. **(I**) Representative images of footprint track. (**J-M)**, For gait analysis, ratio of footprint area of right front paw to left front paw (RF/LF, **J**) and right hind paw to left hind paw (RH/LH, **K**), ratio of footprint intensity of right front paw to left front paw (RF/LF, **L**) and right hind paw to left hind paw (RH/LH, **M**), *p<0.05, **p<0.01, ***p<0.001. Data are shown as mean ± SD.


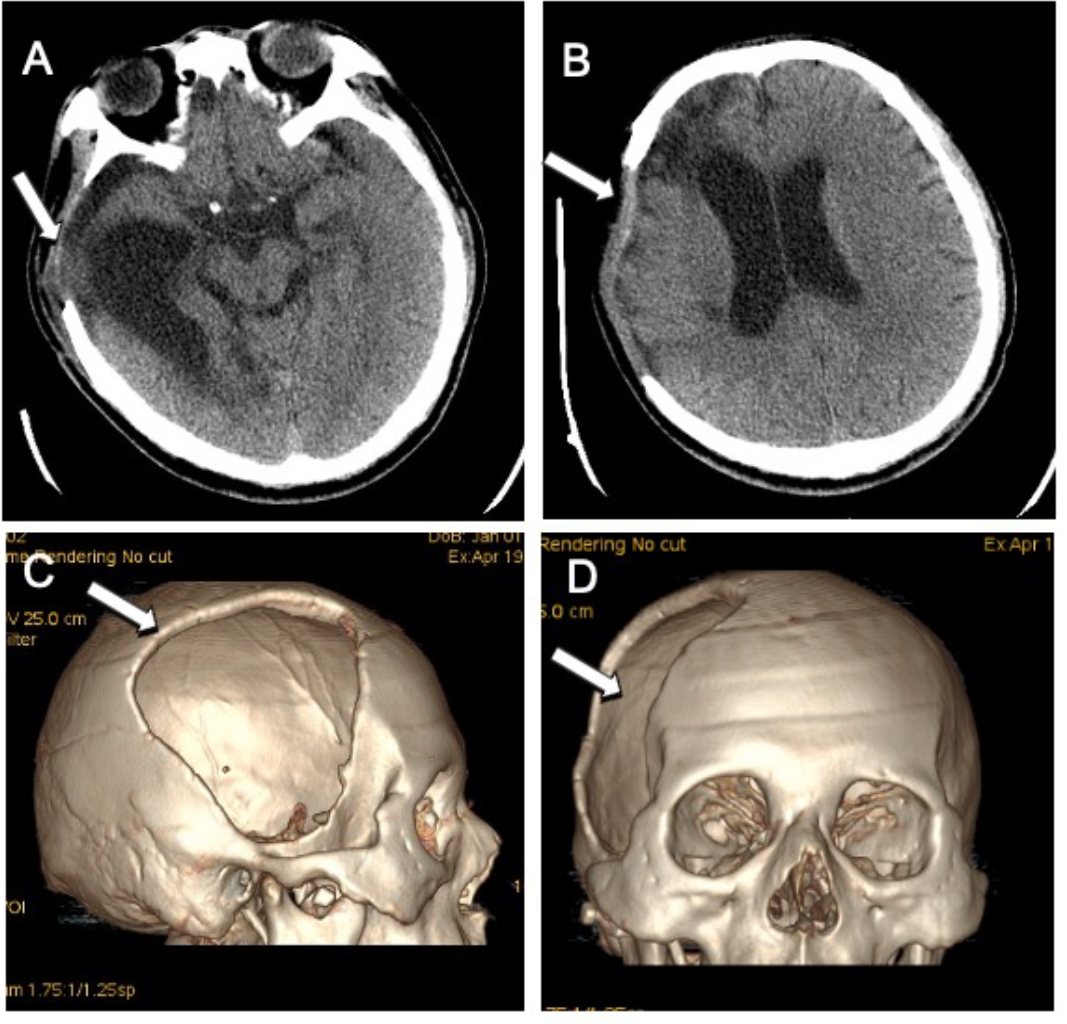


**Supplemental Figure 5 Case 2: A 43-year-old male patient underwent TBI resulting from car accident and was performed decompressive craniectomy**. (**A-B**) Representative CT Image of horizontal position indicated large defects in temporal region of right skull. (**C**) CT 3D reconstruction showed a bone defect in the right skull. (**D**) Anterior view of CT 3D reconstruction.


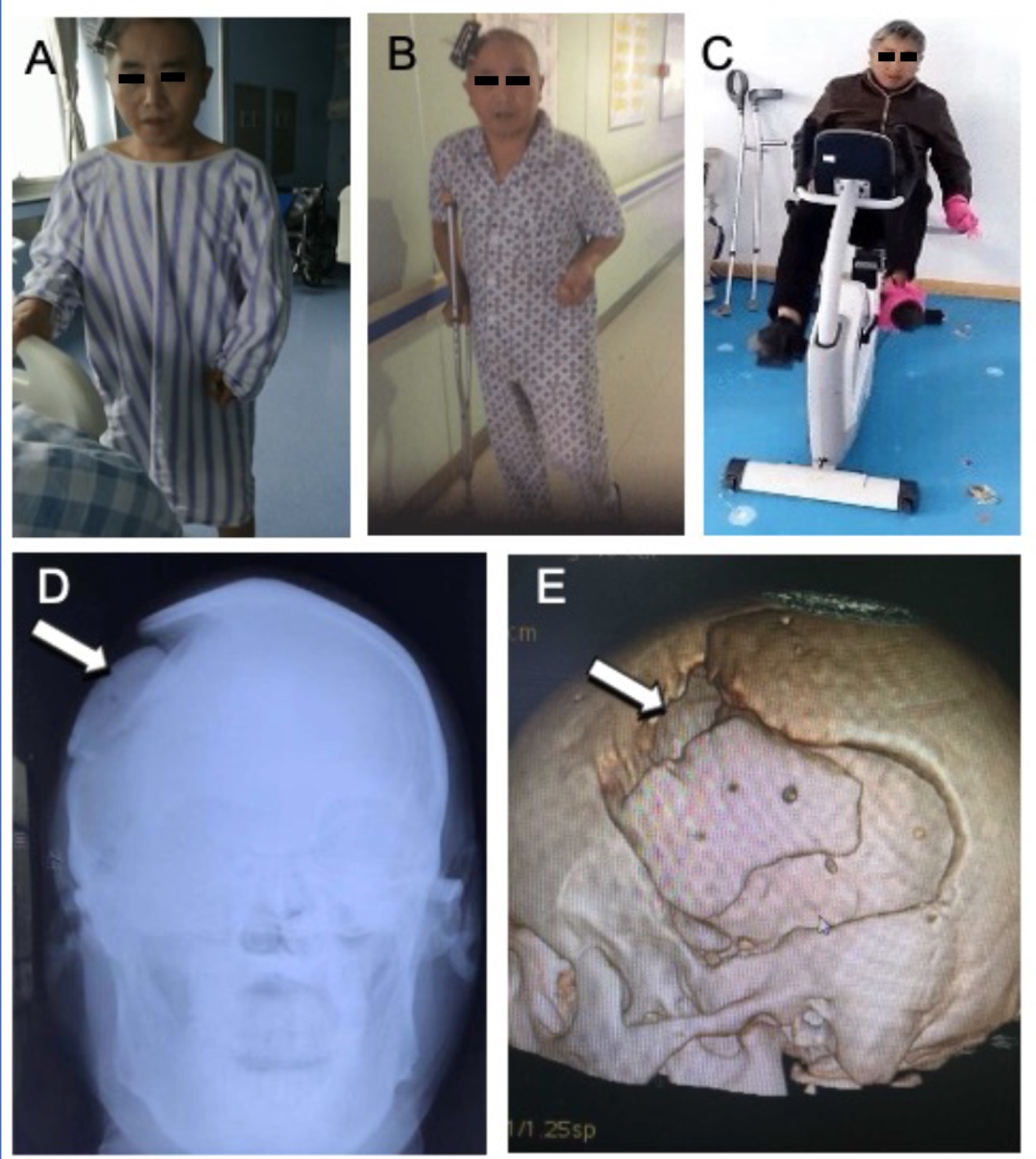


**Supplemental Figure 6** **Case 2: Cranial bone transport ameliorated neurological deficits and enhanced bone reconstruction**. (**A**) Patient was able to stand 2 months after operation. (**B**) The man can walk with a walking stick after 3 months. (**C**) Patient was riding a spinning bike 1 year after operation. (**D**) X ray results suggested bone transport repaired the bone gap. (**E**) CT 3D reconstruction displayed the area of defects were significantly decreased.

Case 2: A 43-year-old male patient underwent TBI caused by car accident. After decompressed craniectomy, CT Images of horizontal position indicated large defects in the temporal region and parietal region of right skull (Supplemental Figure 5A and B). CT 3D reconstruction results showed a calvarial defect was made in part of right temporal and parietal bone (Supplemental Figure 5C and D). He had a left hemiplegia resulting from injury to the right side of the brain, so his hands and legs felt heavy, tense and stiff, he also lacked control of movements of left arms and legs and sustained weakness of muscle of left body. Therefore, he cannot take care of himself and need support of families.

After medication and physical treatment for several months, patients’ neurological deficits were not significantly improved, we intended to perform cranial bone transport to eliminate the negative consequences. Since the right part of skull underwent osteotomy, we designed a bone flap in the rim closed to sagittal suture of the skull, and then there was a bone window to transport the bone flap moving from top to temporal region. After placement of the external fixator and screwing all the pins, bone flap was fixed on the removable frame and transported at a speed of 1mm/day after latency period. Transported was terminated when bone flap arrives at the border of the opposite bone gap, then it was fixed tightly to prepare for bone consolidation which lasted for about 3 months.

Patient was able to stand by himself when holding a handrail 2 months after operation (Supplemental Figure 6A). After 3 months, he can walk by himself slowing with a walking stick, indicating the motor function of his left leg was enhanced (Supplemental Figure 6B). After follow-up for a year, he can ride a spinning bike and take good care of his daily life (Supplemental Figure 6C). The X ray and CT showed the area of bone defect was reduced significantly (Supplemental Figure 6D and E).

**Supplementary Table 1 List of primary and secondary antibodies**

| **Antibody** | **Manufacture** | **Cat. No** | **Species, type** | **Dilution used** |
| --- | --- | --- | --- | --- |
| CD31 | R&D Systems | AF3628 | Goat, polyclonal | 10ug/mL |
| LYVE-1 | Abcam | ab14917 | Rabbit, polyclonal | 1:200 |
| GFAP | Abcam | ab7260 | Rabbit, polyclonal | 1:300 |
| Iba1 | Abcam | ab5076 | Goat, polyclonal | 1:300 |
| NeuN | Abcam | ab177487 | Rabbit, monoclonal | 1:300 |
| P-tau | Invitrogen | MN1020 | Mouse, monoclonal | 1:200 |
| OCN | R&D Systems | MAB1419 | Mouse, monoclonal | 1:200 |
| COL-1 | Abcam | Ab270993 | Rabbit, monoclonal | 1:200 |
| anti-rabbit Alexa Flour 488 | Abcam | ab150073 | Donkey, polyclonal | 1:500 |
| anti-goat Alexa Flour 555 | Invitrogen | A-21432 | Donkey, polyclonal | 1:500 |
| anti-mouse Alexa Flour 594 | Invitrogen | R37115 | Donkey, polyclonal | 1:500 |
| anti-mouse Peroxidase Conjugated | Rockland | 610-1319 | Goat, Polyclonal | 1:500 |
| anti-goat Peroxidase Conjugated | Rockland | 611-1302 | Goat, Polyclonal | 1:500 |
